# Biosynthesis mechanisms, antimicrobial effects, and characterization of silver nanoparticles produced by bacterial and actinomycete isolates derived from a gold mine

**DOI:** 10.1186/s12934-026-03109-4

**Published:** 2026-09-30

**Authors:** Heba Taher, Sara Ibrahim, Noha Salem, Rania Sayed, Hesham Abdulla

**Affiliations:** 1 Botany and Microbiology Department, Faculty of Science, Suez Canal University, Ismailia, Egypt; 2 Materials Testing and Chemical Surface Analysis Lab, National Institute of Standards, Giza, Egypt

**Keywords:** Silver nanoparticles, biosynthesis mechanisms, *Streptomyces*, characterization

## Abstract

**Background:**

Silver nanoparticles (AgNPs) are widely studied for their antimicrobial properties, and microbial synthesis offers an eco-friendly and scalable alternative to conventional methods. While *Streptomyces* spp. are well-known producers, little is known about AgNP biosynthesis by microorganisms adapted to metal-rich environments such as gold mines, which may represent a largely unexplored source of microorganisms with potential for AgNP biosynthesis. This study investigated the biosynthetic potential of bacterial and actinomycete isolates recovered from the Sukari Gold Mine, Egypt, with a focus on optimizing cell-free extract (CFE) preparation, nanoparticle characterization, and understanding the underlying biochemical contributions.

**Results:**

Out of 57 isolates, three *Streptomyces* strains showed the highest AgNP biosynthesis capability. Among the tested methods, soaking was selected as the preferred CFE preparation approach because it provided comparable AgNP biosynthesis while offering a simpler and more practical procedure than freeze/thaw and sonication. The biosynthesized AgNPs exhibited antimicrobial activity against tested microorganisms, with enhanced performance relative to commercial AgNPs. Antimicrobial assessment was supported by MIC and MBC determinations. Biochemical analysis of the CFE revealed the presence of phenols, proteins, and nucleic acids (DNA and RNA), while FTIR analysis identified functional groups including hydroxyl, amine, aromatic, alkene, and alkyne groups, suggesting their involvement in reduction and stabilization processes. The *napA* gene was detected in all active strains, indicating the potential for nitrate reductase production; however, its role in Ag⁺ reduction appears to be contributory rather than definitive. Characterization using UV–Vis spectroscopy, XRD, FTIR, AFM, HR-TEM, and zeta potential analysis confirmed the formation of stable AgNPs with spherical to irregular morphologies, sizes ranging from 5 to 52 nm, and high colloidal stability (zeta potential up to − 37.3 mV).

**Conclusions:**

These findings highlight gold mine–derived actinomycetes as promising candidates for the green synthesis of stable, bioactive AgNPs and suggest that multiple biomolecules act synergistically during nanoparticle formation.

## Background

Nanotechnology is a rapidly advancing field in modern science, with nanoparticles (NPs) serving as its fundamental building blocks. Nanoparticles are defined as structures with at least one dimension at the nanoscale, and they exhibit unique physicochemical properties compared to their bulk counterparts [1]. Among these, silver nanoparticles (AgNPs) are the most extensively studied due to their high surface area-to-volume ratio, chemical stability, electrical conductivity, and notable catalytic and antimicrobial activities [2, 3]. Consequently, AgNPs have been widely applied in biomedical fields as antimicrobial, anticancer, and antiangiogenic agents, as well as in food preservation, textile coatings, and environmental applications [2, 3].

Nanoparticle synthesis using physical and chemical methods is extremely costly and poses environmental concern because of the use of toxic and hazardous chemicals which limit their applications [4, 5]. Conventional physical and chemical methods for nanoparticle synthesis are often associated with high cost and environmental concerns due to the use of toxic and hazardous reagents [4, 5]. In contrast, biological synthesis has emerged as a promising alternative, offering advantages such as cost-effectiveness, eco-friendliness, simplicity, and scalability. In addition, biomolecules present in biological systems can act as natural reducing, capping, and stabilizing agents, eliminating the need for external chemicals and often enhancing nanoparticle functionality [6].

Bacteria, fungi, yeast, actinomycetes, plant and algal extracts have been explored for AgNPs synthesis [6–8]. Microorganisms, in particular, are considered efficient biofactories capable of synthesizing nanoparticles intracellularly or extracellularly. Intracellular synthesis involves the uptake and reduction of metal ions within the cell, whereas extracellular synthesis occurs outside the cell using biomass, culture supernatant, or cell-free extracts (CFE). Several bacterial genera, including *Bacillus*, *Aeromonas*, *Pseudomonas*, and *Lactobacillus*, have been reported to synthesize AgNPs through both intracellular and extracellular mechanisms [9–12].

CFE is considered an alternative and ideal method for the biosynthesis of Ag NPs. CFE is considered an alternative and ideal approach for the biosynthesis of AgNPs. Many techniques are available for CFE preparation, including soaking, freeze/thaw, and sonication methods [13, 14].

Bacteria and actinomycetes inhabiting harsh and metal-rich environments, such as gold mines, are subjected to strong selective pressure due to elevated concentrations of heavy metals. This selective pressure promotes the evolution of adaptive mechanisms that enhance metal tolerance, including efflux systems, enzymatic detoxification, precipitation, and intracellular sequestration [15, 16]. Such adaptations may also facilitate the reduction of metal ions and the stabilization of nanoparticles, making these environments a valuable yet underexplored source of nanoparticle-synthesizing strains [17, 18]. Microorganisms inhabiting gold mines may have a greater capability to synthesize nanoparticles because they can resist ion toxicity. Consequently, Srinath et al. [19] used Bacillus subtilis isolated from a gold mine in India to synthesize AuNPs.

Actinomycetes, particularly members of the genus *Streptomyces*, are of special interest due to their ability to produce diverse bioactive compounds, high protein content, and capacity to thrive under extreme conditions [20, 21]. These characteristics make them suitable candidates for nanoparticle biosynthesis. Previous studies have reported the synthesis of spherical AgNPs (5–25 nm) by *Streptomyces glaucus* [22], as well as mono- and polydisperse nanoparticles by other *Streptomyces* strains [23, 24].

Despite extensive research, the exact mechanisms underlying microbial nanoparticle biosynthesis remain incompletely understood. It is generally accepted that multiple biomolecules, including proteins, polysaccharides, and enzymes, contribute to both the reduction of metal ions and stabilization of nanoparticles [25, 26]. Enzymes such as oxidoreductases and NADH-dependent reductases have been proposed to play a role by facilitating electron transfer during the reduction of Ag⁺ to Ag⁰ [27, 28]. Among these, nitrate reductase has been frequently suggested as a potential contributor to this process [29, 30].

Bacterial nitrate reductase enzymes are classified into three types according to their cellular location, operation organization, and active site structure: periplasmic nitrate reductase (Nap) located in the periplasmic space; respiratory nitrate reductase (Nar), a membrane-bound protein, and assimilatory nitrate reductase (Nas) a cytoplasmic protein [31, 32].

Although AgNP biosynthesis by *Streptomyces* has been widely reported, microorganisms from metal-rich environments such as gold mines remain comparatively underexplored. In this study, bacteria and actinomycetes isolated from different sites within the Sukari gold mine (Marsa Alam, Egypt) were investigated for their ability to biosynthesize AgNPs. The study aimed to evaluate different methods for preparing cell-free extracts, characterize the synthesized nanoparticles, and explore the biochemical and molecular factors contributing to their formation. The antimicrobial activity of the biosynthesized AgNPs was assessed against *Escherichia coli* (NCMB 11943), *Staphylococcus aureus* (ATCC 25923) and *Candida albicans* (ATCC 10231). Nanoparticles were characterized using UV–Vis spectroscopy, X-ray diffraction (XRD), Fourier transform infrared spectroscopy (FTIR), atomic force microscopy (AFM), high-resolution transmission electron microscopy (HR-TEM), and zeta potential analysis.

## Methods

### Isolation of microorganisms

To validate the hypothesis proposing a link between microbial resistance to silver and the biosynthesis of AgNPs, isolates were obtained from the Sukari Gold Mine, Marsa Alam, Egypt. It has been reported that gold mines contain silver, thereby increasing the likelihood of finding silver-resistant isolates [33]. Soil samples were collected during winter 2021 from three different locations/stages within the Sukari Gold Mine, Marsa Alam, Egypt: float tail, final tail, and pool sediment. Samples were collected in sterile polyethylene bags, tightly sealed, transported to the laboratory on ice, and stored at 4 °C until isolation. The isolated microorganisms were subsequently preserved for further investigations. The measured Ag concentrations were 16.7, 26.5, and 60.51 mg/kg, while Au concentrations were 5.64, 7.07, and 27.18 mg/kg in the float tail, final tail, and pool sediment samples, respectively. Soil temperature and pH were not measured at the time of sampling.

A 10 g sample was mixed with 90 ml of sterile saline and stirred at 150 rpm for 15 min and serially diluted. Actinomycetes and bacteria were recovered using four different isolation media: R2A [34], starch casein agar [35], starch casein agar diluted to one-tenth strength, and nutrient agar [36]. The antifungal cycloheximide (0.05 g /l) dissolved in ethanol and filter sterilized was added to all media after autoclaving [37]. All media were sterilized by autoclaving at 15 psi for 15 min and the pour plate technique was used for isolation. Duplicate plates were prepared, incubated at 28 °C until colonies appeared; these were subsequently picked, purified, and preserved as slopes at 4 °C [38].

### Silver resistance test

Recovered isolates were evaluated for silver resistance by culturing on media supplemented with Ag⁺. Starch-casein agar was used for actinomycetes, while nutrient agar was used for bacterial isolates. A filter-sterilized AgNO₃ solution (Merck, Germany) was added to sterile media to obtain a final concentration of 300 mg/l Ag⁺ prior to plate pouring. A standardized inoculum (10⁵ CFU/10 µl) was spot inoculated onto the plates. Actinomycetes were incubated at 28 °C for 7 days, while bacterial isolates were incubated at 37 °C for 24 h. Growth on Ag⁺-supplemented media was compared with control plates lacking Ag⁺ to assess resistance. The experiment was conducted in duplicate.

### Screening of the recovered isolates for AgNPs biosynthesis

All recovered isolates, including both resistant and sensitive isolates, were individually inoculated into flasks containing sterile broth media: nutrient broth for bacteria and starch-casein broth for actinomycetes. The cultures were then incubated with continuous shaking for 24 h (bacteria) and 4 days (actinomycetes). The biomass was separated from metabolites by cooling centrifugation at 4 °C for 5 min at 7000 rpm for bacteria and 5000 rpm for actinomycetes. Subsequently, 10 ml of the metabolite solution was added to 90 ml of Ag^+^ solution in distilled water to achieve a final concentration 50 mg/l Ag^+^, along with a control (10 ml of each medium + Ag^+^ solution). The biomass was washed with sterile distilled water and centrifuged again under the same conditions; then 1 g (wet weight) was suspended in 100 ml of 50 mg/l Ag^+^ solution. All flasks were prepared in duplicate and shaken at 100 rpm at ambient temperature, with 50 mg/l Ag^+^ solution being used as a control. The color change from colorless to brown was visually observed, and UV-Vis spectroscopy measurements were carried out on a spectrophotometer (PG instrument-T60) operated in the range of 300–700 nm at a resolution of 1 nm [39]. Absorption spectra were plotted and λ_max_ was determined using Origin Pro 8.5 software. AgNP concentration was measured by atomic absorption spectrophotometer (Perkin Elmer). The most active isolates were selected based on UV–Vis Scan results and AgNP concentration.

### Synthesis methods

Extraction of AgNPs from biomass and metabolites requires cell lysis and other treatments to eliminate cell remains and the organic matrix of the medium, which are not favored by large-scale production; however, the use of a cell-free extract (CFE) overcomes these concerns [40]. The most active isolates were selected and used to synthesize AgNPs by CFE obtained through different methods. One gram of wet-weight biomass was suspended in 50 ml of distilled water, and three methods were employed to obtain the CFE: soaking, freeze/thaw, and sonication. For the soaking method, the suspension was incubated at room temperature with shaking at 100 rpm for 24 h. The freeze/thaw approach involved freezing the suspension overnight, followed by thawing at 37 °C; this cycle was repeated twice. For the sonication method, the suspension was treated using an ultrasonic probe operating at 50 kHz for 15 min, with 5 min intervals, and cooled at 4 °C before further sonication. After extraction, the CFE was separated by cooling centrifugation at 4 °C and 10,000 rpm for 15 min. Subsequently, 50 ml of CFE from each method was adjusted to 100 ml with deionized water containing AgNO_3_ to achieve a final concentration of 1 mM AgNO_3_. The synthesis conditions used in the present study were selected based on previously published optimization experiments and additional unpublished preliminary experiments, including evaluation of AgNO₃ concentration. The selected conditions, including 1 mM AgNO₃, were subsequently applied throughout the present study [41]. Color development was monitored visually, UV–Vis spectra were recorded after two days. A silver ion solution and the cell filtrate without silver were used as controls. The most efficient extraction method was selected based on UV–Vis scan results and subsequent antimicrobial activity, to further investigate both the synthesis mechanisms and the characterization of the synthesized AgNPs.

### Purification of the biosynthesized AgNPs

To remove unreacted Ag⁺ ions, different purification approaches were evaluated using AgNPs synthesized from soaking CFE of a representative isolate. These included dialysis using a dialysis membrane (Sigma) with a 1.2 kDa molecular weight cut-off at 37 °C overnight, as well as centrifugation at 15,000 rpm for 30, 60, 90, and 120 min under both cooling and non-cooling conditions. The effectiveness of purification and the recovery of AgNPs were assessed by comparing UV–Vis spectral scans before and after treatment. The persistence of the characteristic absorbance peak indicated nanoparticle stability. In contrast, a colored supernatant after centrifugation indicated incomplete sedimentation of AgNPs and ineffective purification. The concentration of AgNPs was quantified using an atomic absorption spectrophotometer (PerkinElmer).

### Antimicrobial activity of the biosynthesized AgNPs

The antimicrobial activity of biosynthesized AgNPs was examined against Gram-negative *Escherichia coli* (NCMB 11943), Gram-positive *Staphylococcus aureus* (ATCC 25923), and the yeast *Candida albicans* (ATCC 10231) using the well diffusion method [42]. Test organisms were cultured in nutrient broth at 37 °C with shaking (100 rpm) for 24 h, followed by centrifugation at 7000 rpm for 5 min. The inocula were adjusted to a 0.5 McFarland standard (approximately 1*10⁸ CFU/ml), and 100 µl of each culture was spread onto Mueller–Hinton agar plates. Wells (6 mm diameter) were prepared using a sterile cork borer. AgNP suspensions were previously quantified by atomic absorption spectrophotometry and standardized to ensure consistent dosing. Each well was loaded with 50 µL containing 50 µg of AgNPs. For each treatment, four wells were used, distributed across two plates. Control experiments included cell-free extract (CFE) without Ag⁺ as a negative control, tetracycline (30 µg/disc) as a positive control, and commercially available AgNPs (the same concentration as the biosynthesized) as a reference for comparison. The plates were kept at 4 °C for 20 min to allow diffusion, then incubated at 37 °C for 24 h. The plates were photographed, and the inhibition zones were measured in mm using ImageJ ij152 software.

The minimum inhibitory concentration (MIC) of AgNPs synthesized using soaking CFE of the most efficient isolate was determined by the broth dilution method against *E. coli* (NCMB 11943) and *S. aureus* (ATCC 25923) (1 × 10⁸ CFU/ml). After incubation at 37 °C for 24 h, MIC was defined as the lowest concentration showing no visible growth. The minimum bactericidal concentration (MBC) was determined by subculturing aliquots from non-turbid tubes onto nutrient agar plates. The lowest concentration that resulted in no colony formation after incubation was recorded as the MBC. All experiments were conducted in duplicate.

### Biosynthesis mechanisms

Various theories on NP biosynthesis have been reported; however, the exact mechanism remains unclear due to the involvement of multiple cellular components and pathways. The literature suggests that biomolecules in the CFE play crucial roles in reducing Ag^+^ to AgNPs and stabilizing them through capping [43]. Subsequently, biochemical, FTIR and molecular analyses of the CFE were conducted.

### Biochemical analysis of CFE

Biomolecules present in CFE act as reducing and capping agents including enzymes, proteins, carbohydrates, and phenolic compounds [44]. Carbohydrates and reducing sugars were qualitatively detected by Molisch’s and Fehling’s tests, respectively [45, 46]. Quantitively, total phenols were measured using a photometer (LF2400), while total proteins, DNA and RNA were measured using NanoDrop spectrophotometer (Thermo Scientific NanoDrop One C, USA). To determine nitrate reductase activity of a modification of the method described by Rider and Mellon [47] was followed.

### Fourier transform infrared spectroscopy (FTIR) of CFE

FTIR spectroscopy serves as a crucial tool for exploring surface chemistry and identifying the functional groups of biomolecules. FTIR analysis was performed directly on the dried CFE (oven-dried at 50 °C) using FT/IR-4100 type A spectrometer (JASCO, Japan). Measurements were conducted over a wavenumber range of 4000 to 450 cm^-1^ at a resolution of 0.96 cm^-1^.

### Molecular analysis

Two enzymes were targeted: membrane-bound respiratory nitrate reductase (narG) and periplasmic nitrate reductase (napA). The salting-out method was used to isolate genomic DNA [48]. The nitrate reductase genes *narG* and *napA* were detected using nested PCR. Detection experiments were performed on DNA of the selected isolates. The *narG* gene was amplified by using *narG* T37 (5’ CAYGGNGTNAAYTGYACNGG 3’) and *narG* T39 (5’ TARTGNGGCCCANCCNCCNCC 3’) primers in the first PCR, followed by *narG* W9 (5’ MGNGGNTGYCCNMGNGGNGC 3’) and *narG* T38 (5’ ACRTCNGTYTGYTCNCCCCA 3’) primers in the second (nested) PCR. In the second run, a targeted amplicon of 385 bp was considered positive [49]. The *napA* gene was amplified using *napA* V16 (5’ GCNCCNTGY MGNTTYTGYGG 3’) and *napA* V17 (5’ RTGYTGRTTRAANCCCATNGTCCA 3’) primers in the first PCR followed by *napA* V66 (5’ TAYTTYYTNHSNAARATHATGTAYGG 3’), and *napA* V67 (5’ DATNGGRTGCATYTCNGCCATRTT 3’) in the second PCR. Similarly, a 385 bp amplicon in the second run was considered positive [50]. Amplification was performed in 0.2 ml thin-walled tubes under the following thermocycling conditions: pre-denaturation at 94 °C for 5 min; 30 cycles of denaturation at 94 °C for 1 min, annealing at 58 °C for 1 min, and extension at 72 °C for 1.5 min; followed by a final extension at 72 °C for 10 min. Five microliters of each PCR product, along with a 1 kb DNA ladder were loaded on a 2% agarose gel (15 × 20 cm) containing 0.5 µg ml^–1^ ethidium bromide. Electrophoresis was run at 110 V for 30 min in freshly prepared 1× TAE buffer using a Bio-Rad PowerPac 300 power supply. After electrophoresis, the gel was checked for bands on a UV-illuminator and photographed.

### Identification of active isolates

The active isolates were identified morphologically and using 16 S rRNA gene sequence analysis. Microscopic characterization was performed on cultures grown on ISP4 medium [51]. The salting-out method was used to isolate the genomic DNA [48]. Two universal primers 27 F and 1522 R were used to amplify the 16 S rRNA gene [52]. Phylogenetic analyses were performed, and maximum likelihood, and bootstrap values were determined on the basis of 1,000 replications via MEGA 11 software.

### Characterization of the biosynthesized AgNPs

Silver NPs were characterized by a UV-vis spectrophotometer at room temperature as described in Sect.  2.3. Structural analysis was carried out by X-ray diffraction (D8 Discovery-Burker Company) over the range of 20º-80º, step of 0.04° and 2 s per step to confirm the biosynthesis of silver nano-sized particles. The functional groups on the surface of Ag NPs were investigated by Fourier transform infrared spectrophotometer (JASCO, FT/IR-4100typeA). The size and shape of nanoparticles were investigated by the nanometrological technique, atomic force microscopy (AFM, 5600LS Agilent, USA), and high-resolution transmission electron microscope (HR-TEM, Jeol JEM 2100, Japan). The stability of nanoparticles was defined by Zeta potential that was measured using Zetasizer Nano ZS90 (Malvern, United Kingdom).

### Statistical analysis

Data were presented as mean ± standard deviation. Prior to statistical analysis, the assumptions of normality and homogeneity of variances were evaluated using the Shapiro–Wilk normality test and Levene’s test, respectively. Analysis of differences in inhibition activity among the AgNPs synthesized by the three extracts against the tested microorganisms was performed using one-way analysis of variance (ANOVA) followed by Tukey’s post hoc multiple comparison test, using the Minitab 17 program, with a significance level set at *p* < 0.05. In addition, the antimicrobial activity of the AgNPs biosynthesized using soaking CFE was statistically compared with that of the chemically synthesized AgNPs for *Escherichia coli* and *Staphylococcus aureus*. For *Candida albicans*, where the chemically synthesized AgNPs produced no inhibition zone, a two-sample Welch’s t-test was used to compare the biosynthesized and commercial AgNPs, with statistical significance set at *p* < 0.05.

## Results

### Isolation of microorganisms

**Fig. 1:**
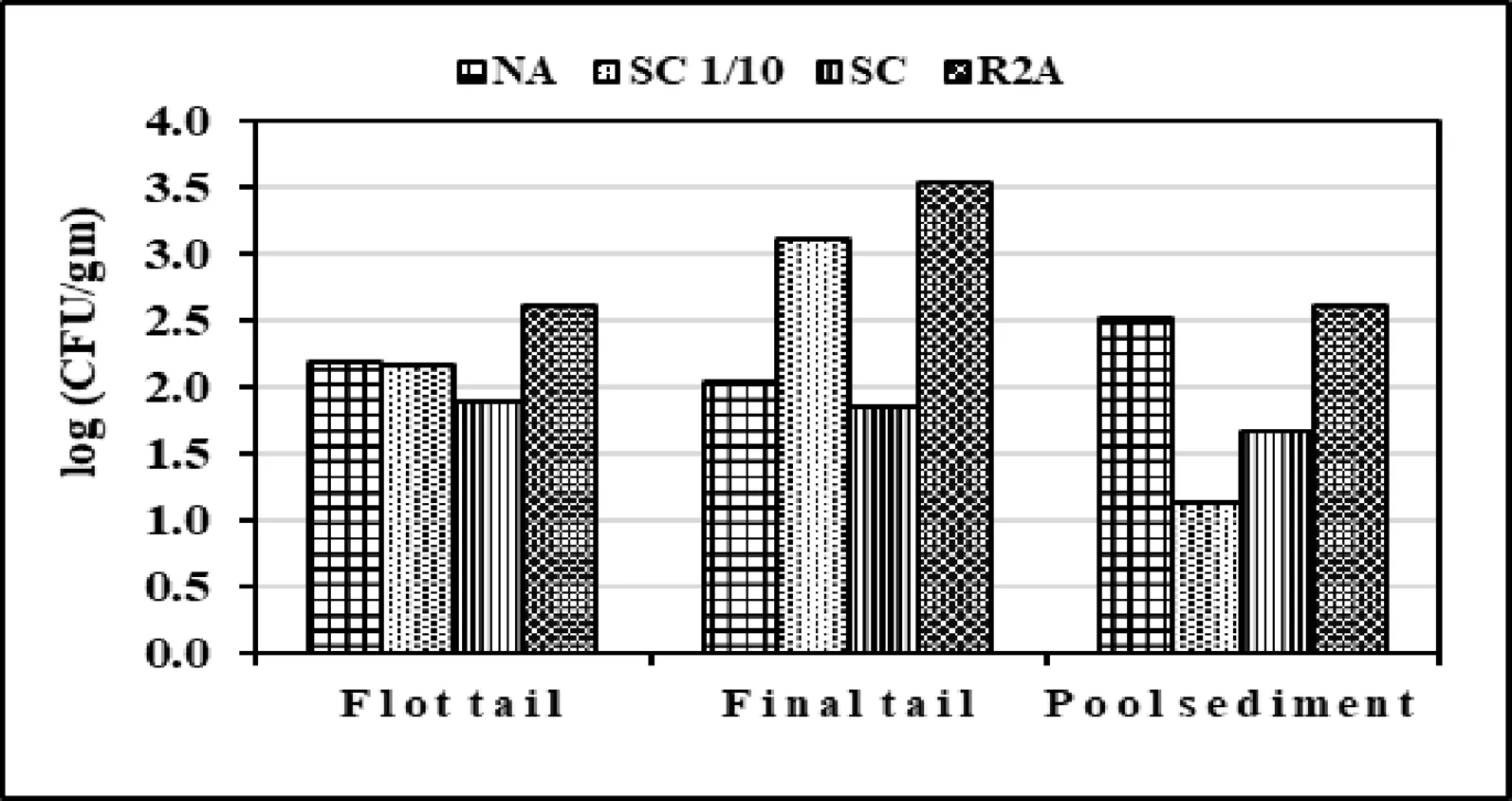
Counts of bacteria and actinomycetes isolated from three samples from Sukari Gold Mine on four different media; Nutrient Agar (NA), Starch Casein 1/10 strength (SC), Starch Casein (SC), and R2A

**Fig. 2:**
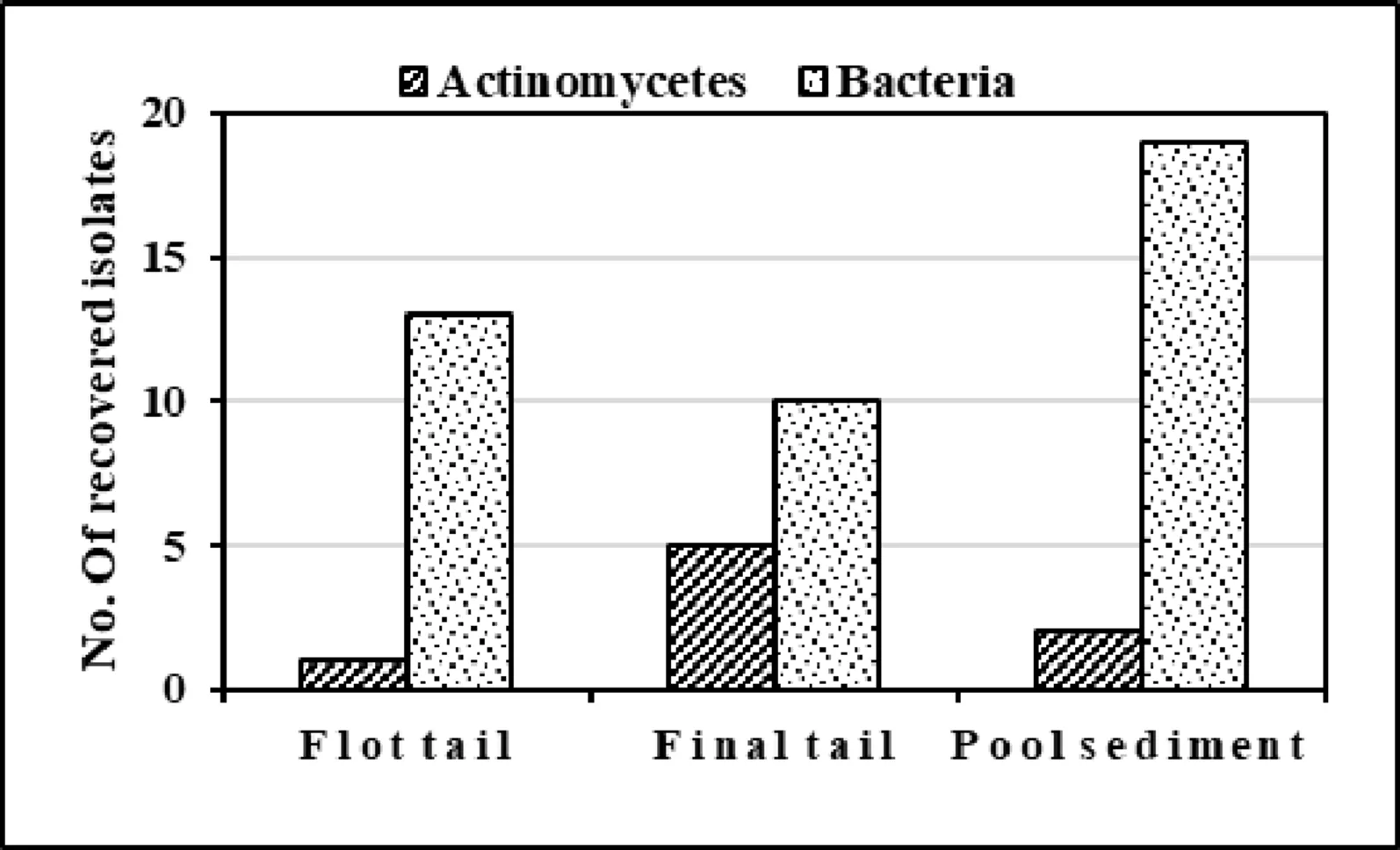
Numbers of actinomycetes and bacteria recovered from the examined samples for subsequent investigations

The results revealed that the highest total count of bacterial and actinomycete isolates was recovered from the final tail on starch casein (1/10) and R2A media as shown in Fig. 1. Based on the morphological characteristics of colonies, the total number of recovered isolates was 57; 15 actinomycetes and 42 bacteria. The highest bacterial count was recovered from pool sediment, while the final tail had the highest actinomycetes count (Fig. 2).

### Silver resistance test

Most of the isolates were sensitive to Ag^+^ at the tested concentration, while only 10 isolates were resistant with weak growth, 6 bacteria and 4 actinomycetes.

### Screening of the recovered isolates for AgNPs biosynthesis

**Fig. 3:**
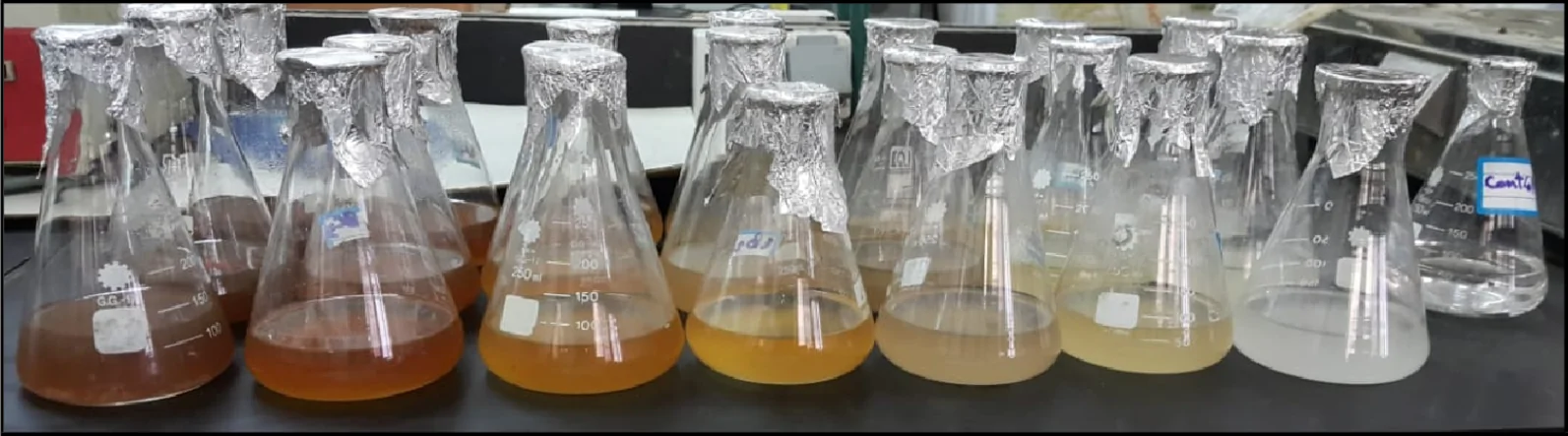
Biosynthesis of AgNPs and color change of silver solution into brown using the recovered bacterial and actinomycete isolates

**Fig. 4:**
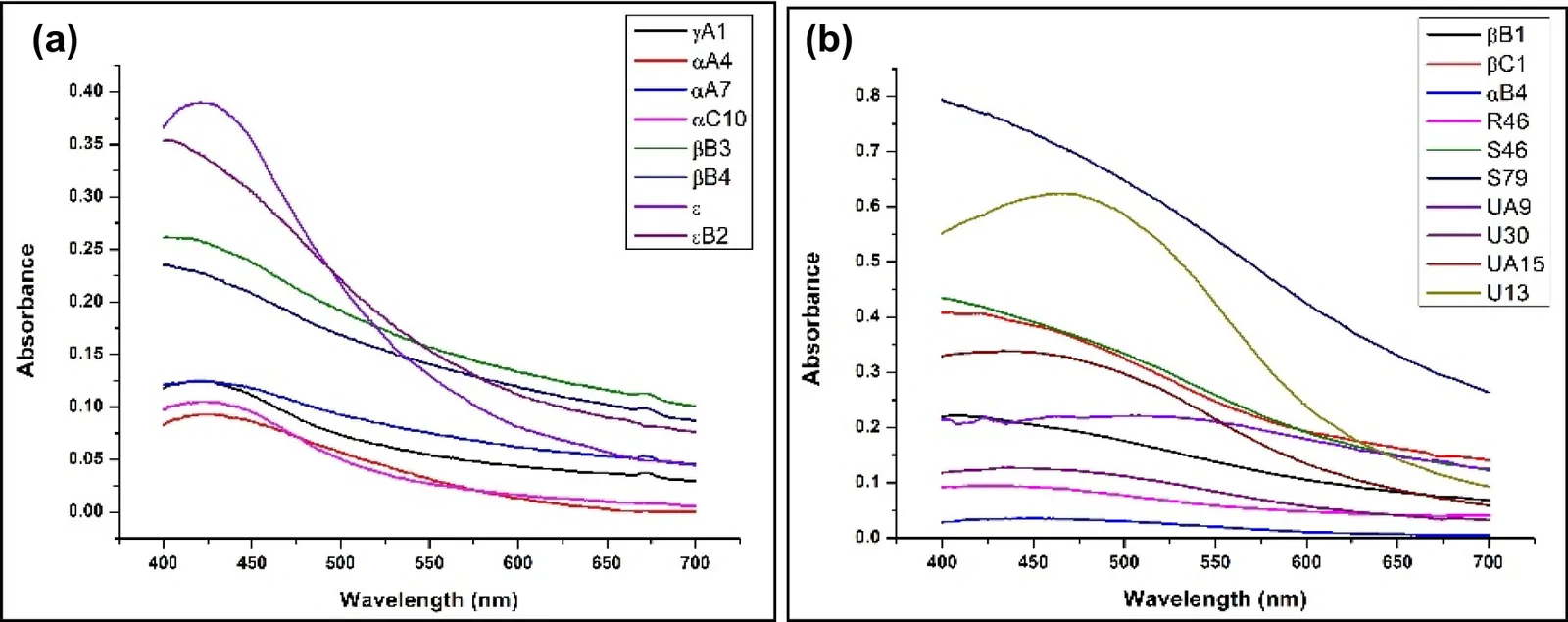
UV–Vis Scan of AgNPs formed by bacterial isolates (**a**) and actinomycetes (**b**), from Ag^+^ concentration (50 mg/l)

The formation of AgNPs was distinguished through the visible observation of the change in color to brown, the characteristic color of AgNPs and no change in control flasks (Fig. 3). AgNPs synthesis using metabolites was excluded, the control of medium changed the color of the solution to brown and only the results of biomass were considered. The formation of AgNPs was confirmed by UV–Vis scan in the range of 300–700 nm (Fig. 4a and b). Isolates that showed peaks at 390–470 nm were considered positive and there were 18 isolates: 8 bacteria (Fig. 4a) and 10 actinomycetes (Fig. 4b). Results of the UV–Vis scan varied among the positive isolates in their peaks (λ_max_) (Table 1), height and tail smoothing (Fig. 4a and b). Among the recovered isolates, the majority were capable of AgNPs synthesis being sensitive to Ag^+^ (12 isolates). From the resistant isolates, the 4 resistant actinomycetes and only two bacteria synthesized AgNPs.

According to the measurement of the biosynthesized AgNPs concentrations, the tested actinomycete isolates synthesized more AgNPs than bacteria. Three actinomycete isolates were considered the most active isolates (βB1, βC1, and βA15) selected depending on both UV–Vis scan results and the concentration of the biosynthesized Ag NPs. They showed well-defined absorption peaks (λ_max_) appeared at 410–435 nm and formed the highest AgNPs concentration (Table 1).

**Table 1:** The Concentration and λ_max_ of AgNPs synthesized by the positive bacterial and actinomycete isolates, the highlighted rows refer to the selected active isolates

	Isolates	Concentration (mg/l)	λ_max_
Bacterial Isolates	γA1	1.97	417
	αA4	7.8	420
	αA7	2.6	413
	αC10	5.41	417
	βB3	7.1	402
	βB4	9.9	400
	εC1	7.9	421
	εB2	4.05	401
Actinomycetes	βB1	24.9	423
	βC1	14.6	413
	αB4	4.5	455
	R46	11.96	410
	S46	6.9	400
	S79	6.29	400
	UA9	5.56	425
	U30	8.2	433
	βA15	14.04	434
	U13	9.2	461

### Synthesis methods

**Fig. 5:**
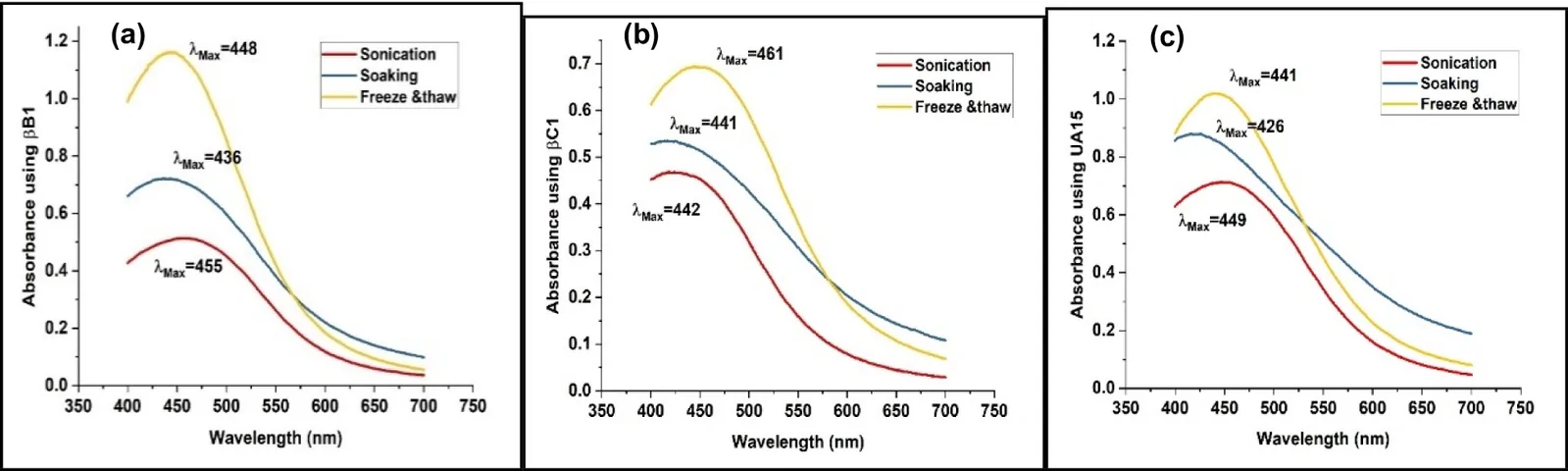
UV–Vis Scan of AgNPs synthesized by different methods, Freeze/Thaw, Soaking and Sonication using actinomycete isolate βB1 (**a**), βC1 (**b**), βA15 (**c**)

According to the UV–Vis scan results, the highest absorbance and narrower peaks were obtained for AgNPs synthesized using CFE prepared by the freeze/thaw method (Fig. 5). The λ_max_ values ranged from 440 to 460 nm for the sonication method, 442–455 nm for freeze/thaw, and 426–442 nm for soaking. The three isolates showed comparable UV–Vis profiles within each preparation method, although βB1 showed slightly higher absorbance and favorable λ_max_ values. Although freeze–thaw produced higher absorbance peaks, the results obtained using soaking were comparable. Considering its simpler procedure, lower cost, and fewer processing steps, soaking was selected for subsequent experiments.

### Purification of the biosynthesized AgNPs

**Fig. 6:**
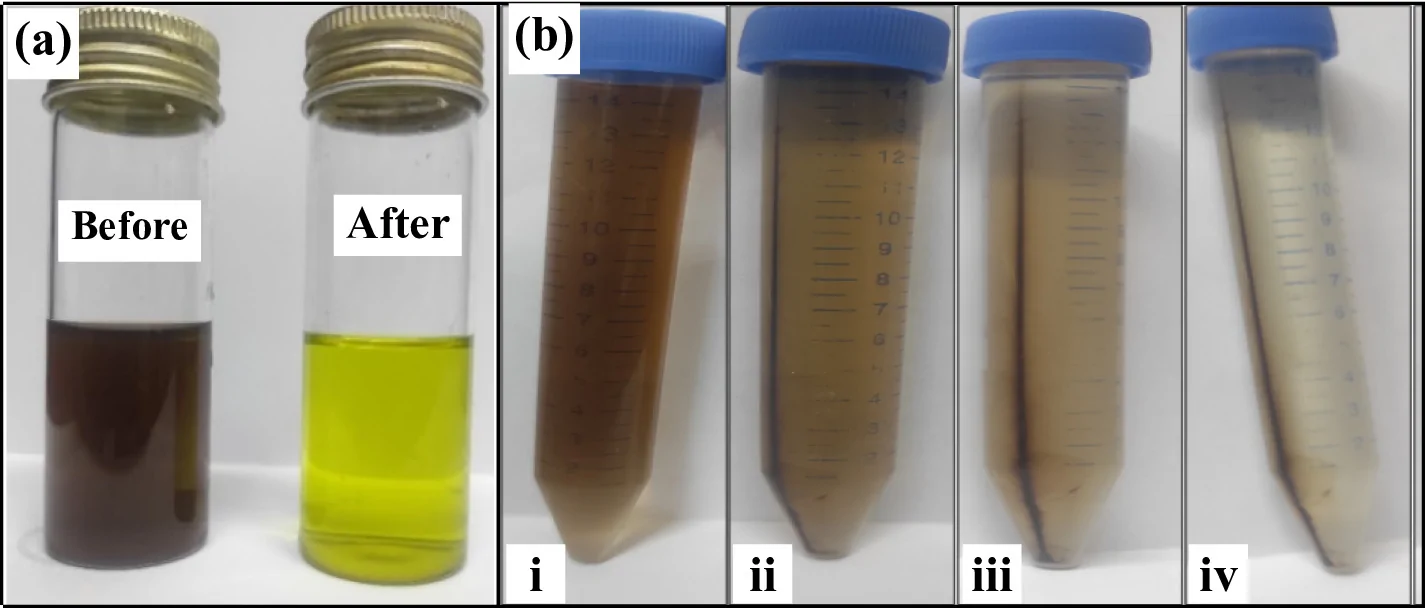
Biosynthesized AgNPs before and after purification using **a** dialysis, **b** cooling centrifugation at 15,000 for (ii) 30, (iii) 60, (iv) 90 min compared to non-purified (i)

Purification using a dialysis sack caused fainting in the brown color of the biosynthesized AgNPs (Fig. 6a). The concentration of purified AgNPs through dialysis was 16.7 ppm, and the λ_max_ shifted from 448 to 400 nm. Cooling and non-cooling centrifugation did not show noticeable differences. During centrifugation, the brown color of the supernatant gradually diminished with increasing centrifugation time, reaching complete clarity after 90 min (Fig. 6b). After centrifugation, λ_max_ was observed at 446 and 435 nm by cooling and non-cooling, respectively compared to non-purified AgNPs with λ_max_ of 448 nm. Convergent concentrations were obtained (15.4 and 15.0 ppm) by cooling and non-cooling centrifugation at 15,000 for 90 min, respectively. Accordingly, cooling centrifugation at 15,000 for 90 min was considered the best purification method.

### Antimicrobial activity of the biosynthesized AgNPs

All the tested microbial strains were inhibited by the biosynthesized AgNPs but with different degrees and the inhibition activity wasn’t found to be isolate-dependent. The diameter of the inhibition zone was found to be between 10 and 37 mm and greater than those of AgNPs synthesized chemically and the positive control (Table 2). Among the tested microbial strains, *Staphylococcus aureus* (ATCC 25923) was the most sensitive, the highest inhibition effect was for AgNPs synthesized by freeze/thaw extract of βA15 isolate while AgNPs synthesized by all extracts of βC1 were the weakest. The inhibition effect was lower on *Escherichia coli* (NCMB 11943) and *Candida albicans* (ATCC 10231), but with all the tested microbes the weakest inhibition was by AgNPs synthesized by βC1. The diameter of inhibition zones against the tested microbial strains due to AgNPs synthesized by soaking extract were the highest except for βA15 isolate against *Staphylococcus aureus* (ATCC 25923).

**Table 2:** Inhibitory behavior of the biosynthesized AgNPs using different synthesis methods by the selected actinomycete isolates compared to synthetic AgNPs and antibiotic Tetracycline (30 µg)

			E. coli	S. aureus	C. albicans
			Diameter of Inhibition Zone (mm)		
CFE			0	0	0
TE 30 µg			25.5 ± 0.73	29.63 ± 1.1	
Commercial AgNPs			13.8 ± 1.01	14.6 ± 1.02	0
Biosynthesized AgNPs	βB1	Freeze-Thaw	17.3 ± 1.35 **c**	33.1 ± 4.44	25.2 ± 5.61
		Soaking	28.6 ± 0.7 **a**	33.7 ± 1.05	21.1 ± 6.6
		Sonication	28.4 ± 1.19 **a**	32.5 ± 0.9	25.7 ± 8.49
		p-value	0.000***	**0.864**	**0.693**
	βC1	Freeze-Thaw	18.9 ± 2.76	21.8 ± 4.14	12.2 ± 0.55
		Soaking	20.4 ± 2.16	23.6 ± 2.28	12.9 ± 0.55
		Sonication	19.2 ± 1.15	20.8 ± 6.51	11.1 ± 0.92
		p-value	**0.677**	**0.765**	0.051*
	βA15	Freeze-Thaw	25.4 ± 0.38 **b**	37.1 ± 0.43	14 ± 0.06 **d**
		Soaking	30.2 ± 0.59 **a**	32.7 ± 0.31	12.6 ± 0.3 **d**
		Sonication	11.6 ± 0.86 **e**	32.2 ± 0.67	16.2 ± 0.43 **c**
		p-value	0.000***	**0.687**	0.000***

Results are means of the inhibition zones diameter (mm)±standard deviation.βB1, βC1 and βA15 are actinomyctes isolates. *E. coli*: *Escherichia coli* (NCMB 11943), *S. aureus*: *Staphylococcus aureus* (ATCC 25923) and *Cand. albicans*: *Candida albicans* (ATCC 10231). CFE: Cell Free Extract TE: Tetracycline. St. D: standard deviation. *, ***; significant at *p* < 0.05, 0.001. Means sharing the same letter are not significantly different according to Tukey’s HSD test (*p* < 0.05)

**Fig. 7:**
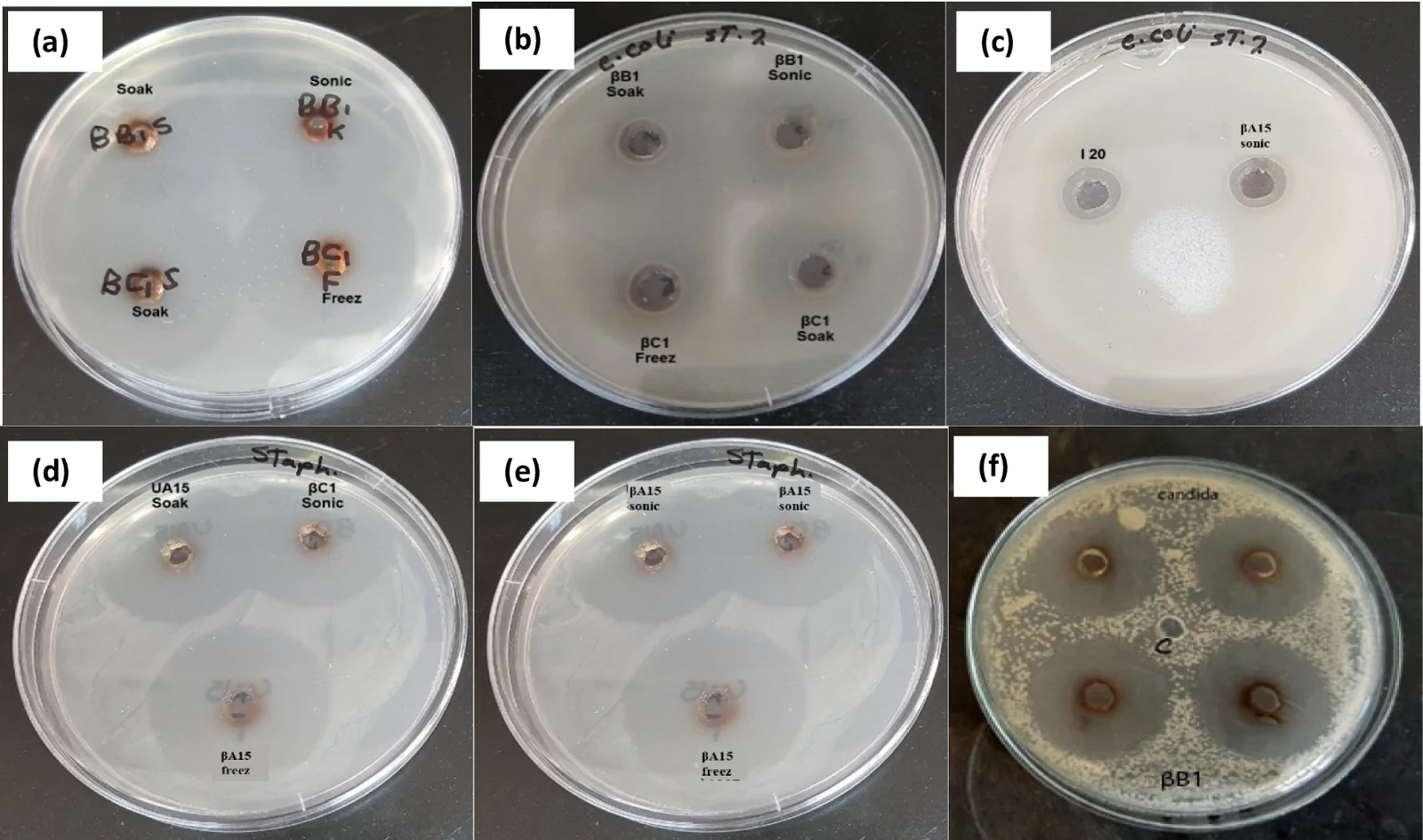
Representative photos of agar plates display the inhibition zones (**a**), (**b**) and (**c**) due to Ag NPs biosynthesized by βB1, βC1 and βA15 against *E. coli*, (**d**) and (**e**) Ag NPs biosynthesized by βC1 and βA15 against *S. aureus* and (**f**) Ag NPs biosynthesized by βB1 against *C. albicans*

Considering the extraction methods, there was no remarkable difference in inhibition activity among most of the AgNPs synthesized by the extracts against each tested microbe. However, a noticeable difference was observed among the AgNPs synthesized by the three extracts of βA15 isolate against *Escherichia coli* (NCMB 11943). Additionally, differences were found in the inhibitory activities of AgNPs synthesized by the three extracts of βB1 isolate against *Escherichia coli* (NCMB 11943) and βC1 isolate against *Candida albicans* (ATCC 10231). Representative photos of the inhibition zones are shown in Fig. 7. For *C. albicans*, the chemically synthesized AgNPs produced no inhibition zone.

The antimicrobial activities were statistically compared with those of commercial AgNPs. The results showed that the biosynthesized AgNPs exhibited significantly greater antimicrobial activity than the commercial AgNPs. The p-value was 0.015 for the comparison against *Escherichia coli* (NCMB 11943) and 0.009 for the comparison against *Staphylococcus aureus* (ATCC 25923). A two-sample Welch’s *t*-test yielded *p* = 0.031, indicating that the biosynthesized AgNPs exhibited significantly greater antifungal activity against *C. albicans*.

The MICs of AgNPs synthesized using the soaking CFE of the βB1 isolate against *E. coli* (NCMB 11943) and *S. aureus* (ATCC 25923) were 20 and 15 µg/ml, respectively, while the MBCs were 40 and 30 µg/ml.

### Mechanisms of AgNPs biosynthesis

The antimicrobial activity of AgNPs synthesized using soaking CFE was the best and it was cost-effective and easy to prepare; thus, it was selected for the subsequent investigation.

### Biochemical analysis of CFE

Carbohydrates and reducing sugars were not detected in the soaking CFE of the three isolates. Quantitative analysis revealed the presence of phenols, nitrate reductase enzymes, proteins, DNA, and RNA (Table 3). The highest concentration of phenols was detected in soaking CFE of isolate βA15, followed by βB1 and βC1. Nitrate reductase enzyme was not detected in βA15 and its concentration in βB1 was higher than that in βC1. The highest concentrations of proteins, DNA and RNA were detected in the CFE of βC1.

**Table 3:** Biochemical qualitative and quantitative analysis for soaking CFE of βB1, βC1 and βA15

	Cell free Extract (CFE)		
	βB1	βC1	βA15
Qualitative analysis			
Carbohydrate	ND	ND	ND
Reducing sugar	ND	ND	ND
Quantitative analysis			
Phenol (mg/l)	0.30	0.1	0.5
Nitrate Reductase Enzyme (mg/l)	0.0062	0.0033	0.000
Total proteins (mg/1)	98	189	81
DNA (mg/l)	7.659	12.204	4.698
RNA (mg/l)	5.618	9.278	3.619

ND: Not Detected

### FTIR of CFE

**Fig. 8:**
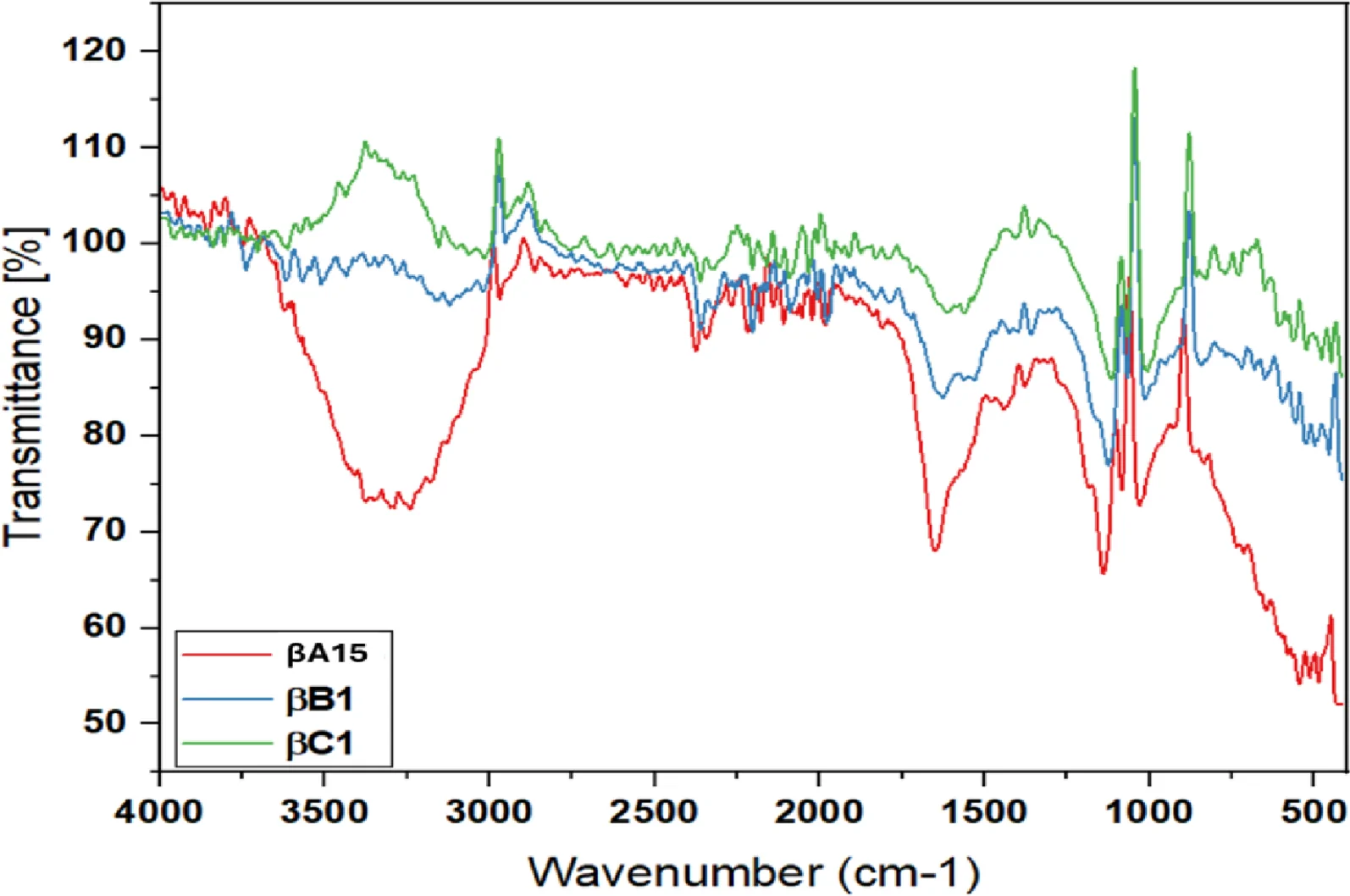
FTIR spectra of dried CFE of the three active isolates

FTIR analysis of CFEs revealed multiple functional groups, as illustrated in Fig. 8. The FTIR spectra of the dried CFEs from isolate βB1 and isolate βC1 exhibited the same functional groups with slight wavenumber variations. Common peaks, including O-H, N-H, C-H, C ≡ C, C = C, N = O, and Cl-I vibrations, corresponding to different chemical groups, such as phenols, proteins, aromatic components, alkyne, alkenes, nitro, and halo components, respectively were observed.

Notably, the FTIR spectra of dried CFE from isolate βA15 displayed two additional peaks, indicating the presence of S-H stretching and S-C ≡ N stretching, corresponding to thiol and cyanate. Detailed interpretations of the FTIR peaks for the dried CFEs of these three strains are presented in Table 4.

**Table 4:** Interpretations of FTIR peaks of dried CFE of βB1, βC1, and βA15

Peak	βB1	βC1	βA15	Bond	Functional group
	Wavenumber (cm^− 1^)				
1, 2	3574 − 3434	3567–3438	3588	O–H stretching	Phenol and alcohol
3, 4	3287	3280	3362–3497	N–H stretching	Aromatic amine
5	3015.7	3015.84	3028.2	C-H stretching	Aromatic ring
6	2949	2954	2970	C-H stretching	Alkene
7	2766.34	2739.8	2750.1	C-H stretching	Aldehyde
8			2572.94	S-H stretching	Thiol
9	2249	2206.26	2204.24	C ≡ C stretching	Alkyne
10			2154	S-C ≡ N stretching	cyanate
11	1638.27	1635.64	1633.61	C = C stretching	Alkene
12	1535.9	1538.05	1519.95	N-O stretching	Nitro compound
13	1319	1260.71	1270.31	C-N stretching	Aromatic amine
14	1386.3	1359.6	1359.6	O-H bending	Phenol and alcohol
15	1067.67	1067.54	1067.86	C-N stretching	Aromatic and aliphatic amine
16	1006.31	1014.32	1014.62	C-F stretching	Halo component (fluro)
17	914.34	951.31	913.11	C-H bending	Alkene
18	887	881	846	C–H bending	Aromatics
19	727.18	720.3	719.68	C-Cl stretching	Halo component (chloro)
20	650.63	682.21	697.65	C–Br stretching	Halo component (bromo)

### Molecular analysis

**Fig. 9:**
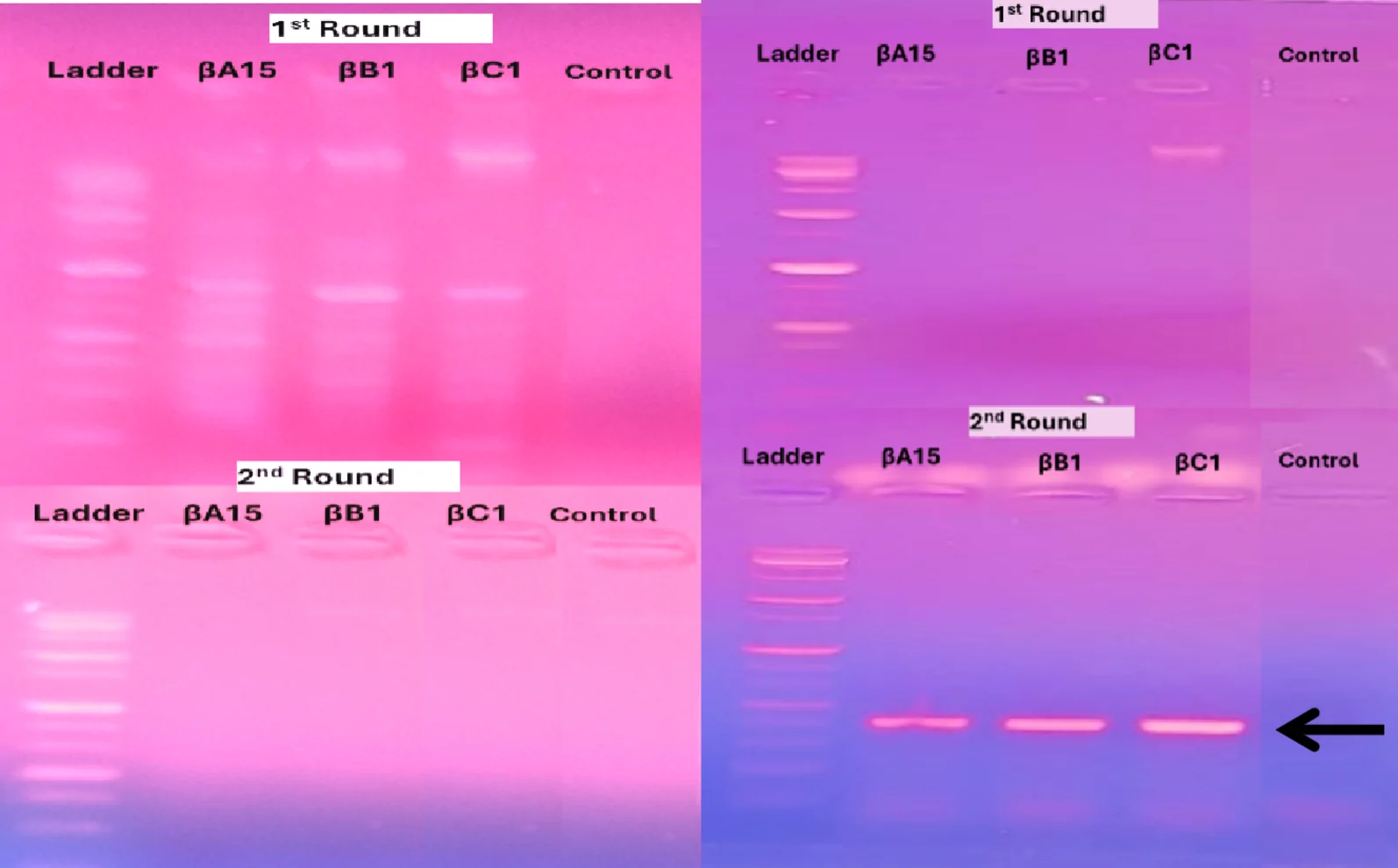
PCR products of *narG* (**a**) and *napA* (**b**) gene compared to 1 kb ladder (left), arrow referred to the targeted amplicon

No specific band for the *nar*G gene was detected in any of the selected isolates in the second round (Fig. 9a). For the *nap*A gene the amplicons of the first round were used as a template for the second PCR round. In the second run, the targeted amplicon with 385 bp size was recorded for all three isolates (Fig. 9b).

### Identification of active isolates

#### Morphological identification

**Fig. 10:**
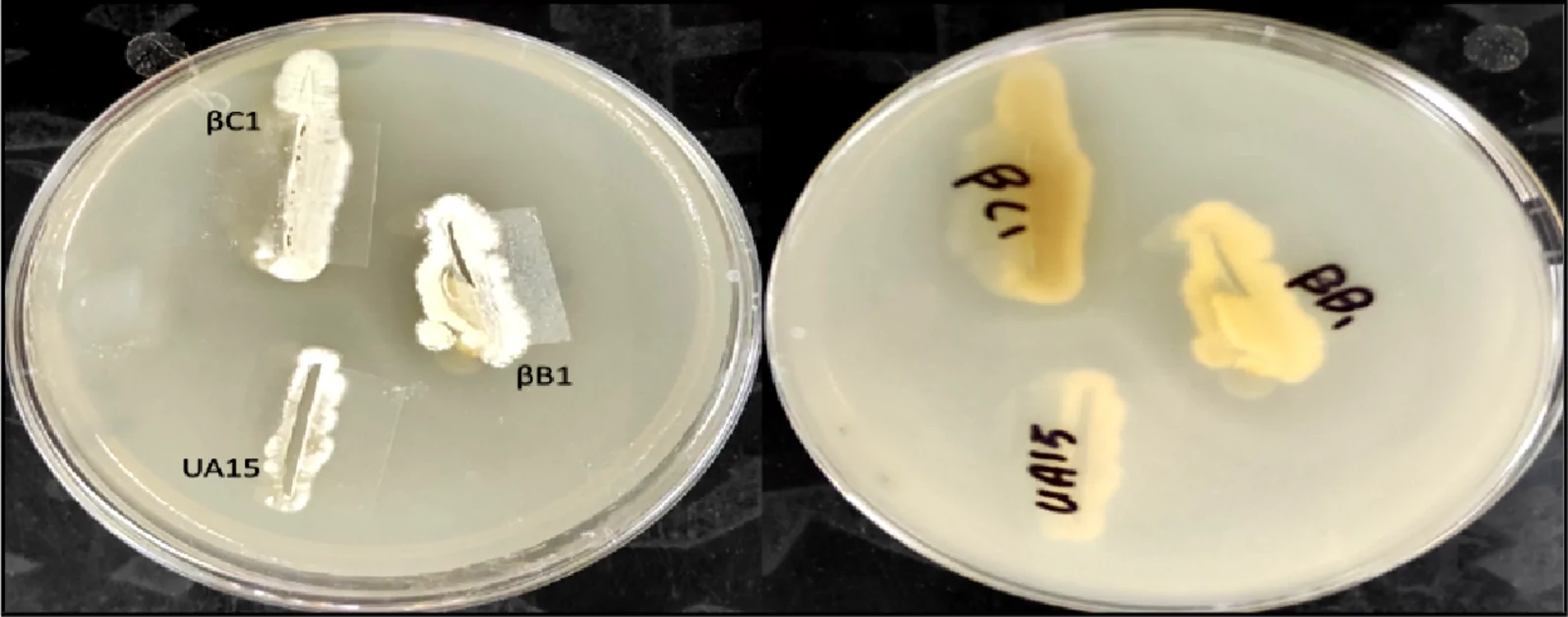
Aerial (left) and substrate (right) mycelia of βB1, βC1 and βA15 on SC agar after 3 days

**Fig. 11:**
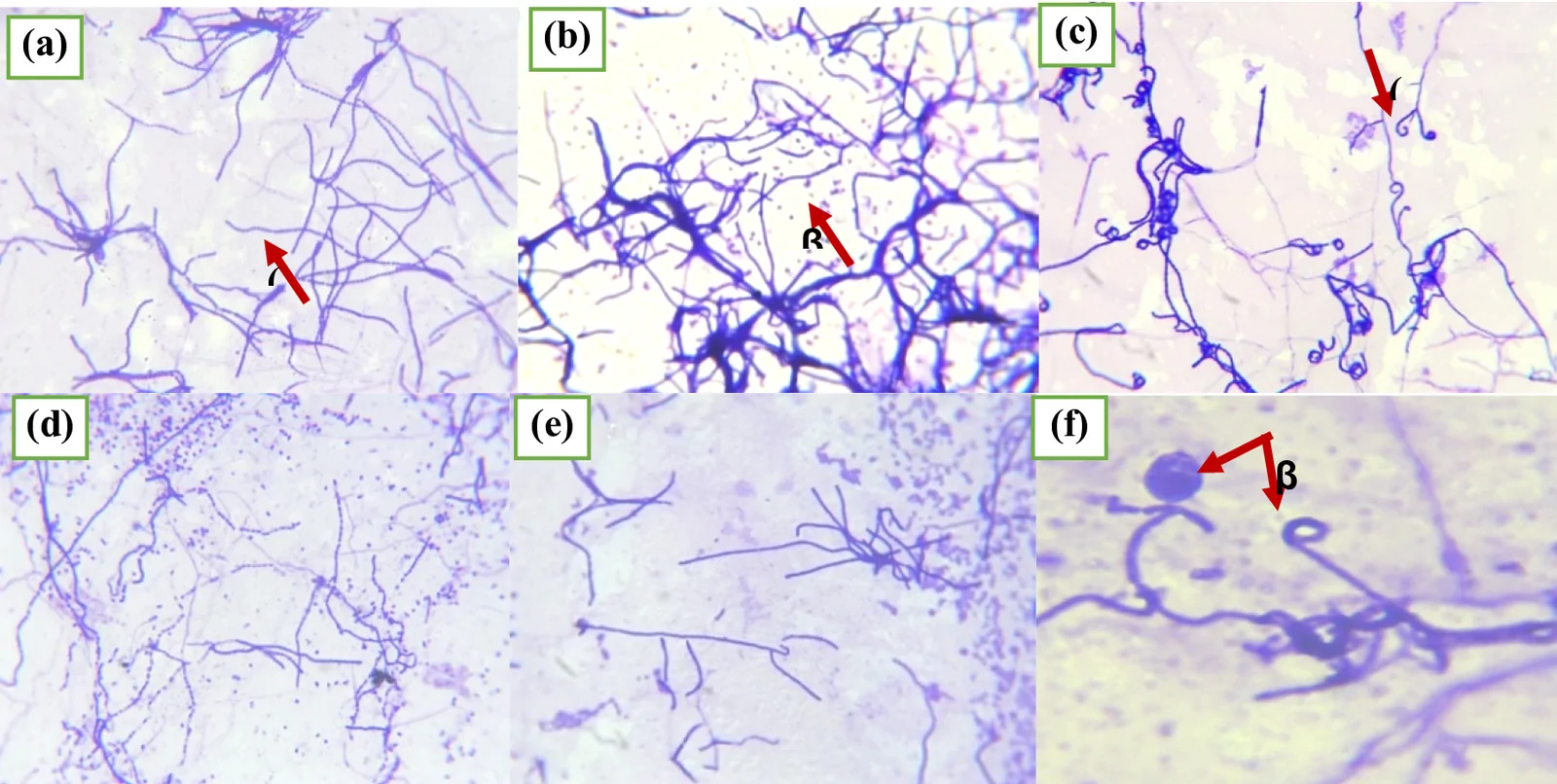
Light microscope (1000X) photomicrograph of βB1 (**a**), βC1 (**b**), βA15 (**c**) after 3 days and βB1 (**d**), βC1 (**e**), and βA15 (**f**) after 14 days. Arrows show spore chains

All isolates formed aerial and substrate mycelia as shown in Fig. 10. The aerial mycelium of βB1 was a long-branched spore chain, βC1 forms flexuous and branched spore chains, while βA15 forms a closed spiral spore chain that developed in a late state. Fragmentation was observed in the three isolates after 14 days (Fig. 11).

### Molecular identification

**Fig. 12:**
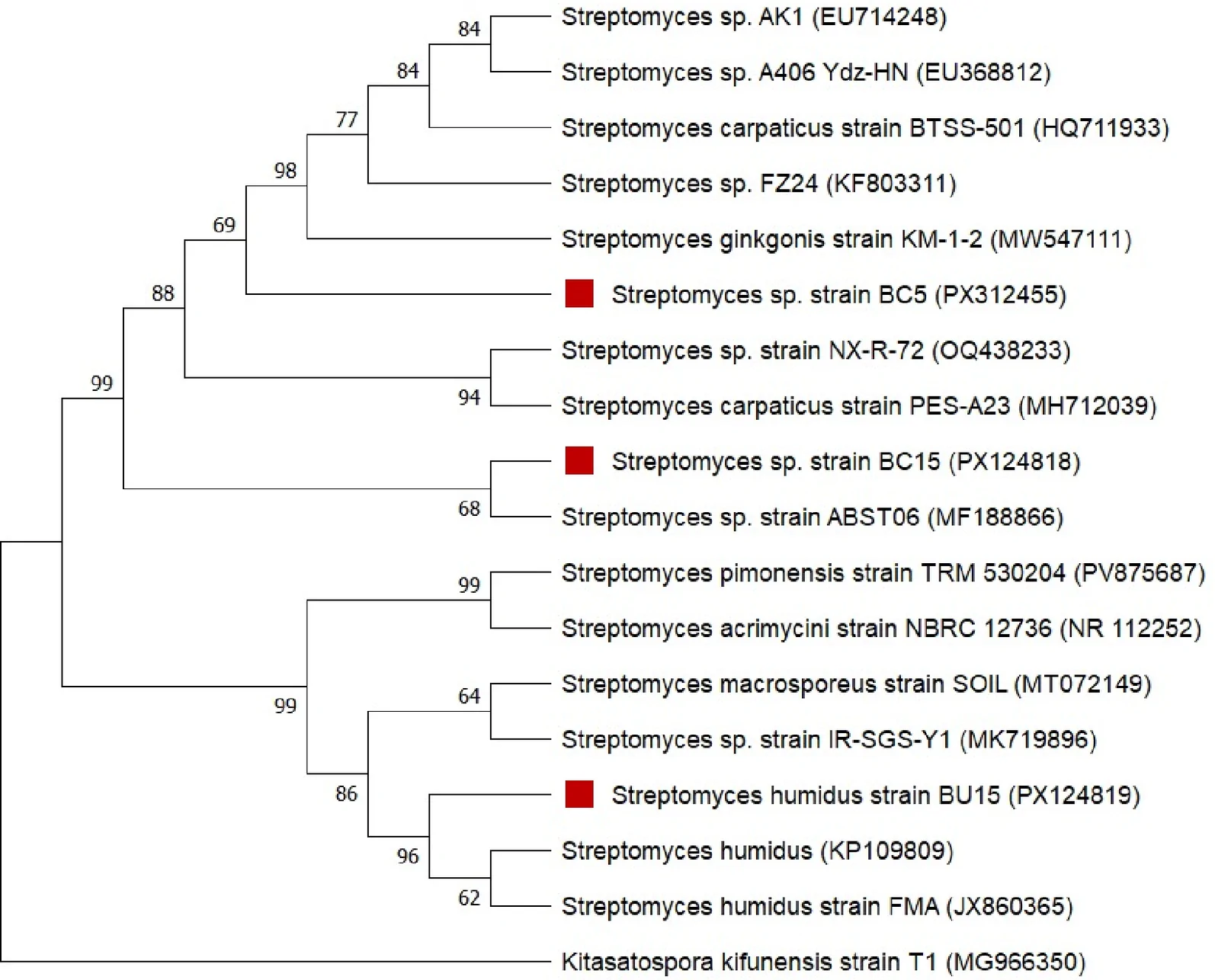
Maximum-Likelihood tree based on 16 S rRNA gene sequences showing the relationships among the selected actinomycete isolates (marked) sequenced and their phylogenetically closest species

The sequences obtained were compared with those deposited in NCBI databases and identified *Streptomyces* sp. for βC1 and βB1 and *Streptomyces humidus* for βA15 (Table 5; Fig. 12).

**Table 5:** 16 S rRNA gene sequence similarity between the most active isolates and the closest strains on the NCBI database

Isolates	Reference strain	Query cover	Percent identity	Accession no.
βA15	*Streptomyces humidus*	100%	99.5%	PX124819
βC1	*Streptomyces* sp.	100%	99%	PX312455
βB1	*Streptomyces* sp. 193,409	100%	99%	PX124818

### Characterization of the biosynthesized Ag NPs

#### X-ray diffraction (XRD)

**Fig. 13:**
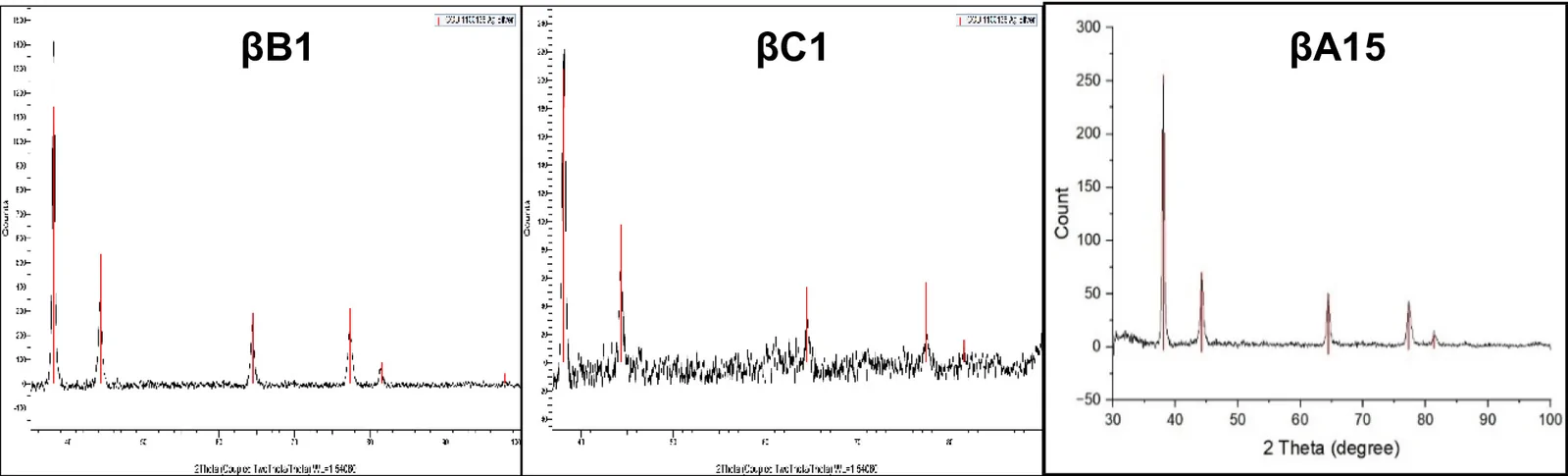
XRD patterns of the biosynthesized AgNPs

The XRD analysis of AgNPs synthesized using βB1, βC1, and βA15 revealed Bragg’s diffraction peaks at 2*ϴ* = 38.12°, 44.31°, 64.46°, 77.42° and 81.56° after comparing with standard XRD spectrum. The observed peaks assigned to the planes (111), (200), (220), (311) and (222) of the face-centered cubic lattice for AgNPs as shown in (Fig. 13). The average crystalline size of the synthesized AgNPs was calculated by Debye-Scherrer’s formula after fitting as a Gaussian distribution. The calculated crystalline size was 20.6, 30.3, 27.2 nm for βB1, βC1, and βA15 respectively.

### Fourier transform infrared spectroscopy (FTIR)

**Fig. 14:**
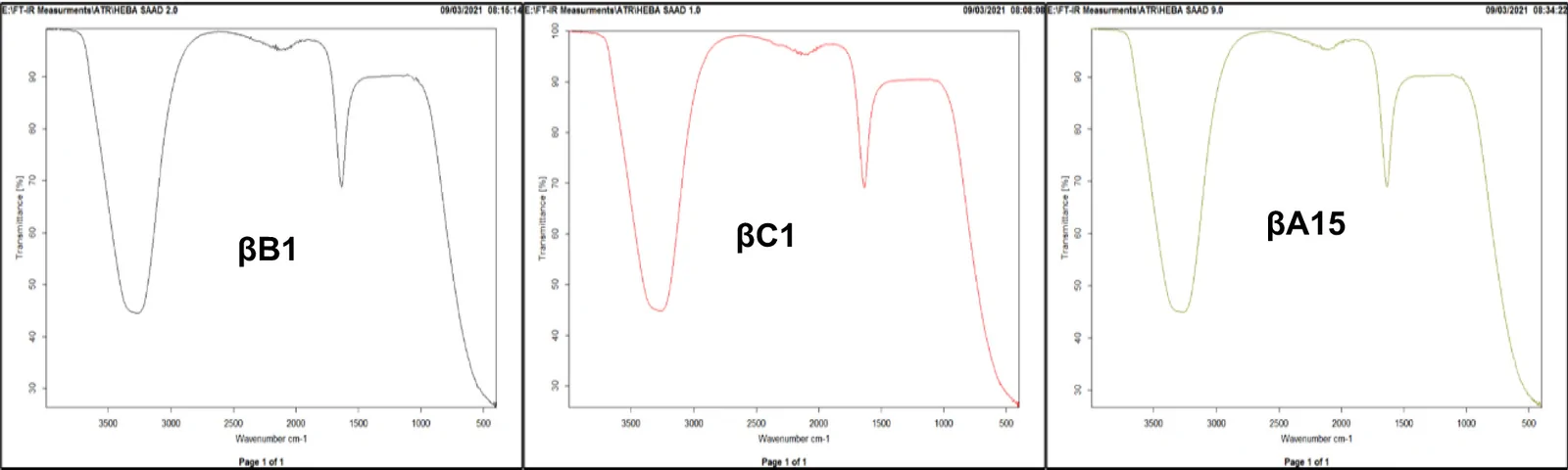
FTIR patterns of the biosynthesized AgNPs

FTIR spectral analysis of AgNPs biosynthesized by βB1, βC1, and βA15 showed absorbance bands at 3470.28, 2096.24, 1639.2, and 529 cm^− 1^ corresponding to O-H, S-C ≡ N, C = C, and C-Br functional groups, representing phenol and alcohol, isothiocyanate, alkene, and bromo halo components, respectively (Fig. 14).

### High-resolution transmission electron microscope (HR-TEM)

**Fig. 15:**
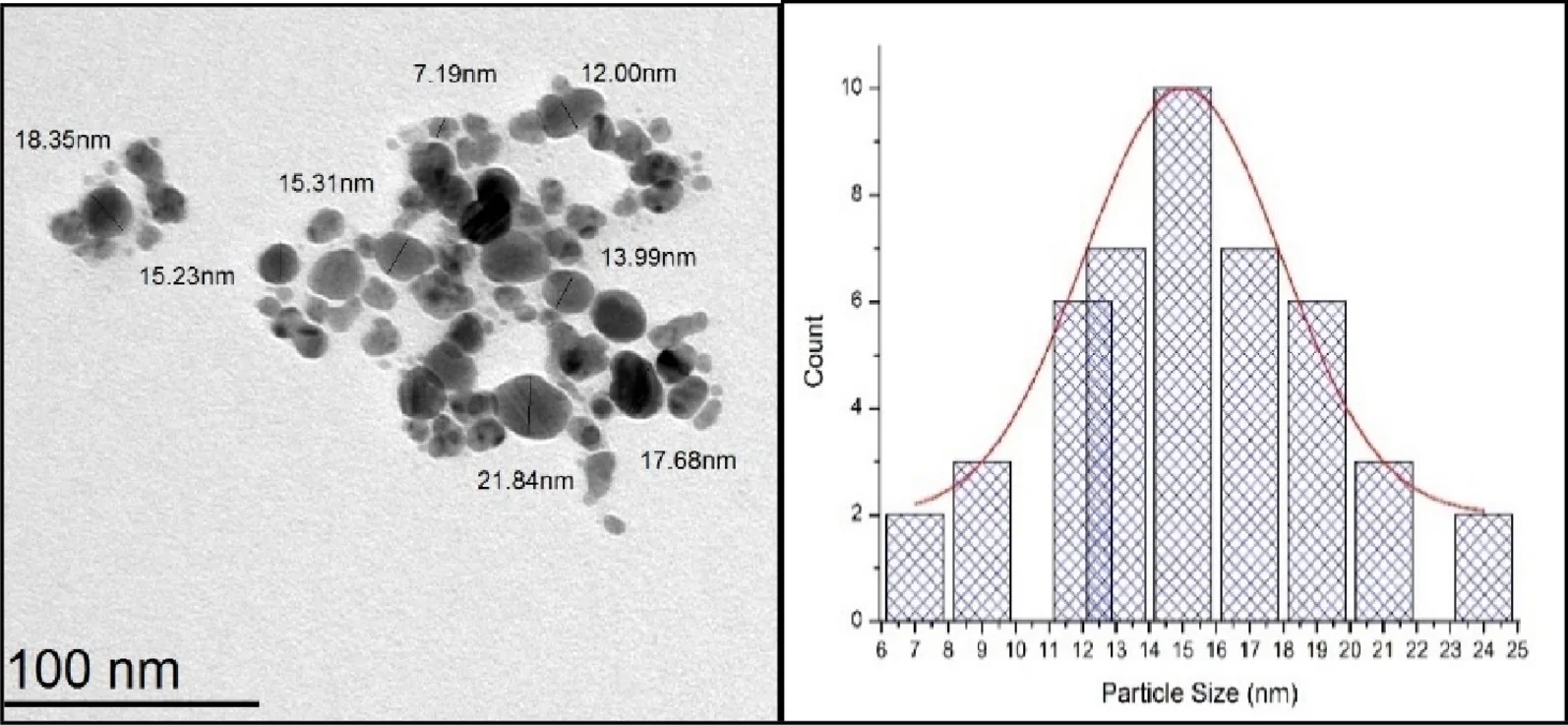
HR-TEM images and particle size distribution chart of biosynthesized AgNPs using βB1, their average size and standard deviation are 15.3 ± 5.3 nm (the total number of NPs counted for particle size distribution were around 60 particles)

**Fig. 16:**
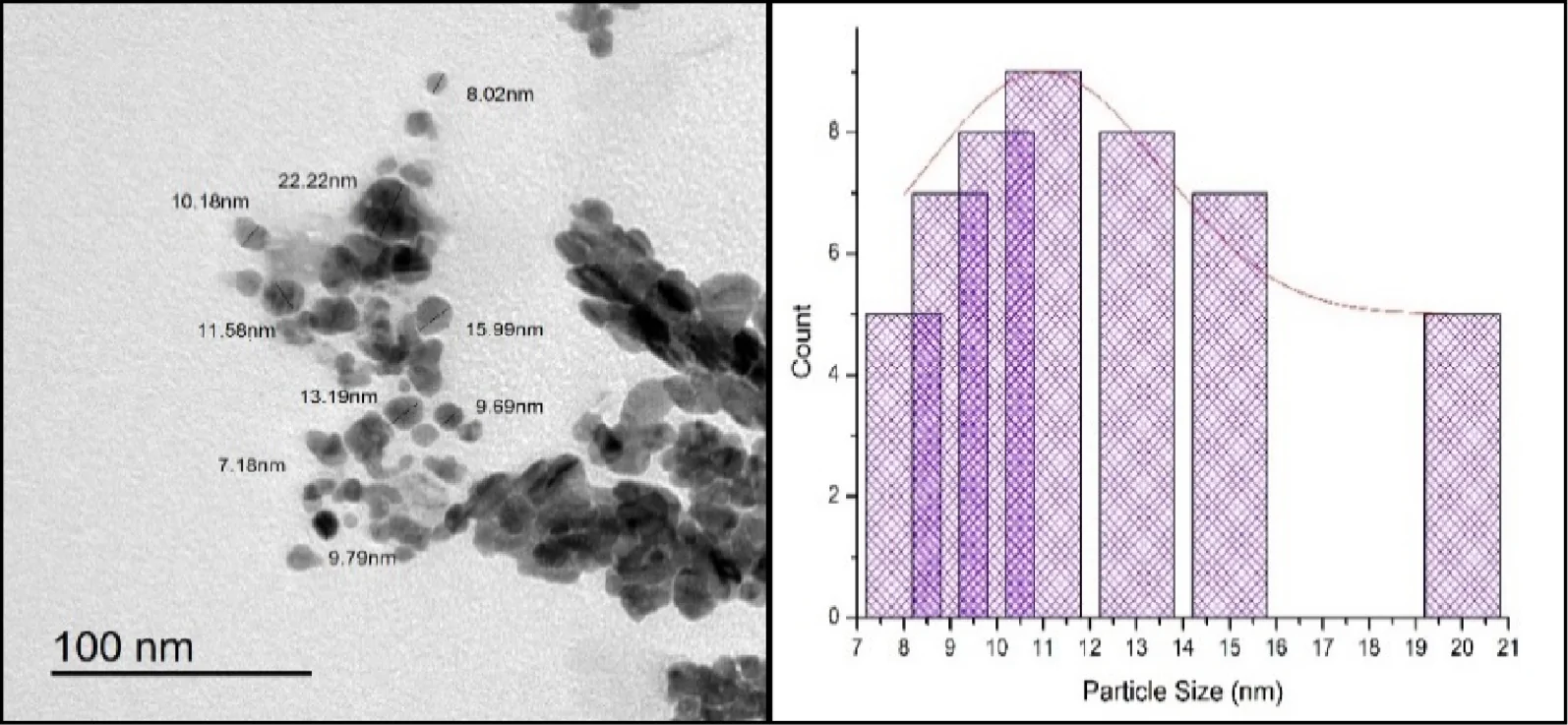
HR-TEM images and particle size distribution chart of biosynthesized AgNPs using βC1, their average size and standard deviation are 11.3 ± 4.7 nm (the total number of NPs counted for particle size distribution were around 60 particles)

**Fig. 17:**
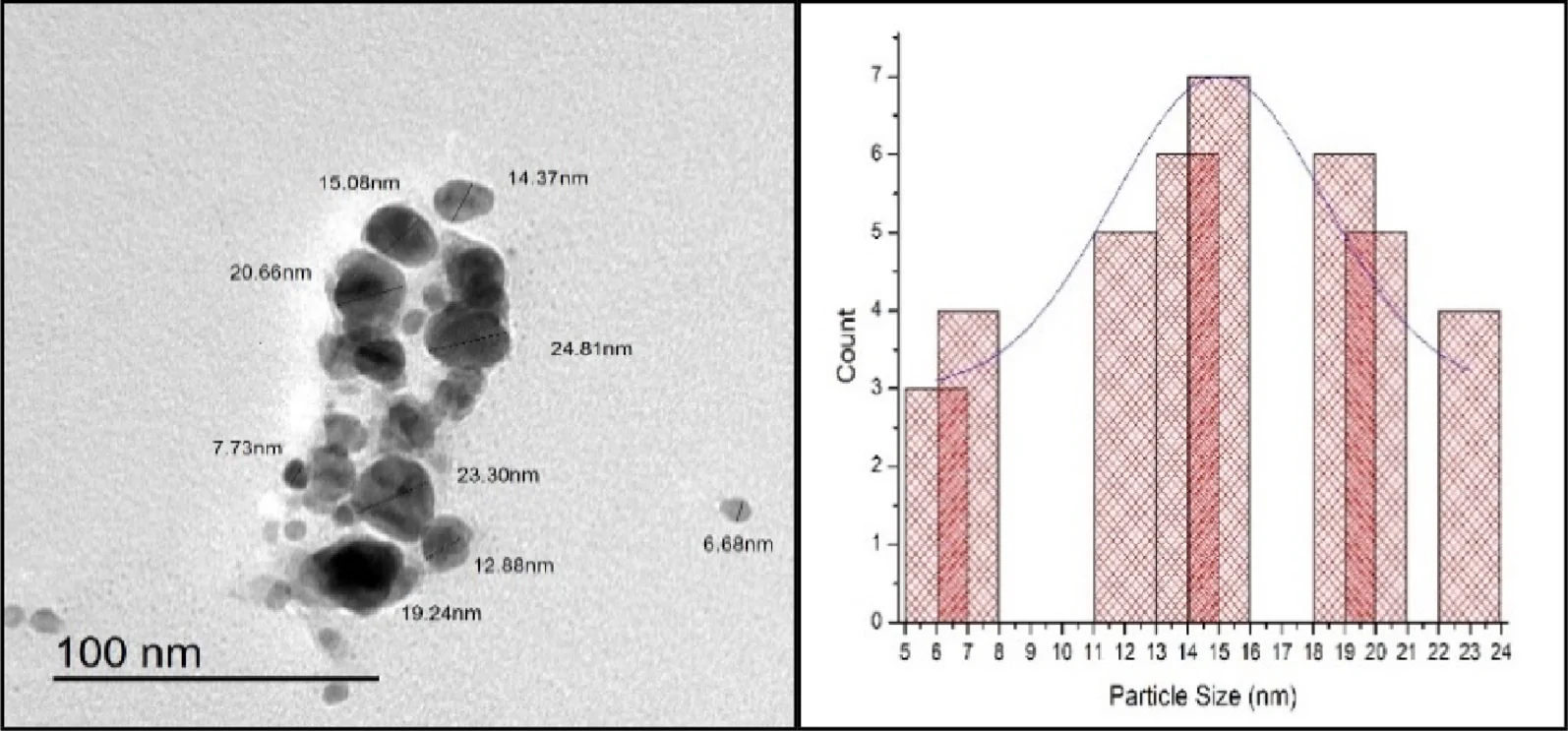
HR-TEM images and particle size distribution chart of biosynthesized AgNPs using βA15, their average size and standard deviation are 15 ± 5.9 nm (the total number of NPs counted for particle size distribution were around 60 particles)

The size of AgNPs was determined by measuring the diameter of the particles on HR-TEM images (Figs. 15, 16 and 17). HR-TEM images of AgNPs synthesized using βB1 revealed that the bio-synthesized AgNPs were well dispersed and spherical in shape. The size ranges from 6 to 25 nm (Fig. 15). HR-TEM images of AgNPs synthesized using βC1 and βA15 revealed that the biosynthesized AgNPs were not well dispersed and had spherical to irregular shapes. The size of the particles ranged from 7 to 21 nm for βC1 and from 5 to 24 nm for βA15 (Figs. 16 and 17).

### Atomic force microscope (AFM)

**Fig. 18:**
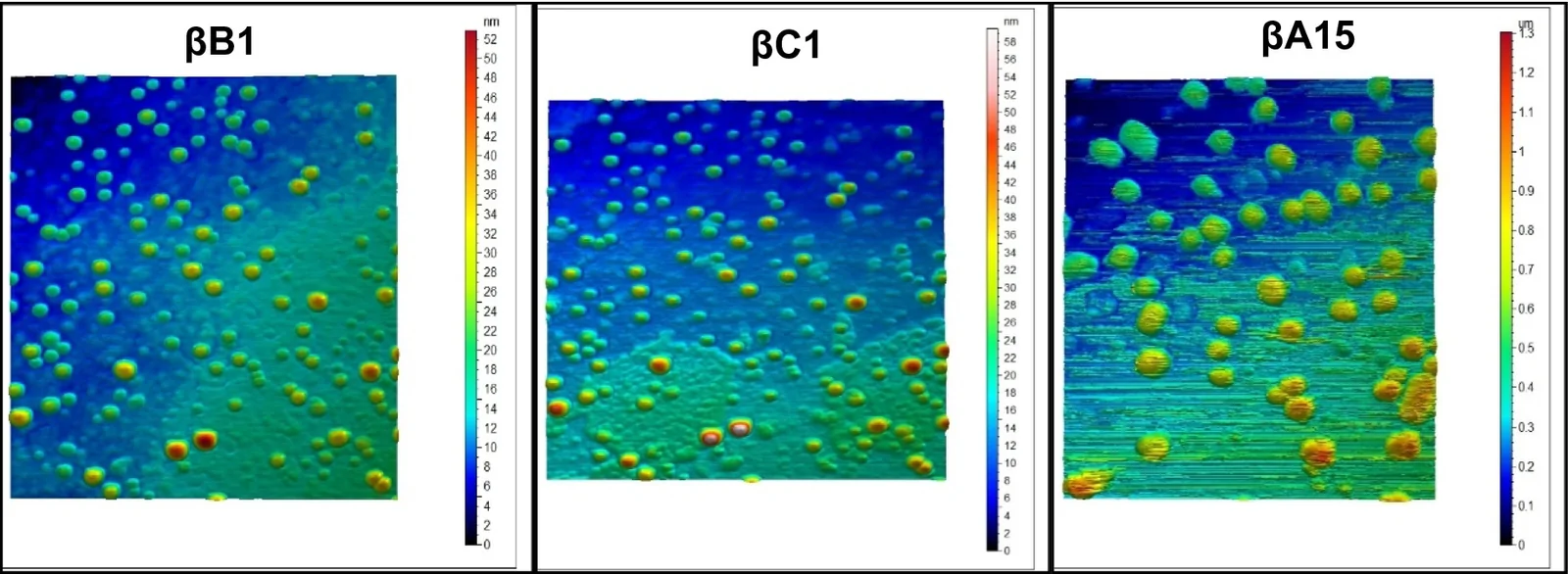
AFM AC Mode of the biosynthesized Ag NPs, the colored bar next to the images represents different heights

Although AFM images showed a larger field than those of HR-TEM, their results confirmed the HR-TEM results. βB1 showed the most AgNPs homogenous suspension while some particles synthesized by βA15 exceeded nanoscale. AFM of AgNPs synthesized by isolates βB1 and βC1 revealed that their size was in the range of 6 to 52 nm, while the range of βA15 was over than 50 nm (Fig. 18).

### Zeta-potential

**Fig. 19:**
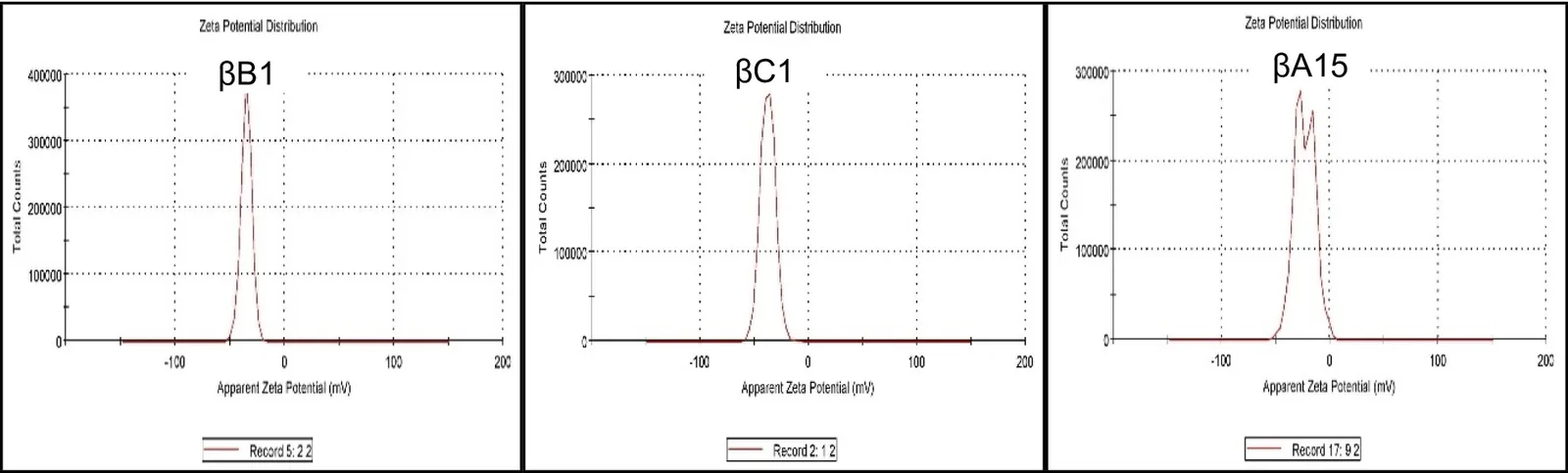
Zeta potential distribution of the biosynthesized Ag NPs

The value of the zeta potential of biosynthesized AgNPs was negatively charged for all isolates. For βB1 the synthesized AgNPs were averred 35.0 ± 4.94 mV, and 37.3 ± 6.76 mV for AgNPs synthesized using βC1, while βA15 was found to be 23.2 ± 5.64 mV (Fig. 19).

## Discussion

Microorganisms inhabiting metal-rich environments are constantly exposed to elevated concentrations of heavy metals, which promote the evolution of mechanisms for metal tolerance and transformation. These adaptations can enhance their ability to convert Ag⁺ into its metallic form [53]. Biosynthesis of AgNPs by *Streptomyces* has been reported, microorganisms isolated from metal-rich environments remain comparatively underexplored. Therefore, investigating the biosynthetic potential of gold mine-derived microorganisms may provide new insights into microbial AgNP biosynthesis and the biochemical factors involved in nanoparticle formation.

In this study, bacterial and actinomycete isolates recovered from a gold mine were investigated for AgNP biosynthesis. A total of 57 isolates were obtained, of which 10 exhibited resistance to Ag⁺. The synthesis of AgNPs was confirmed by a color change from colorless to brown and by UV–Vis absorption peaks in the range of 390–470 nm. Related results were reported by Mukherjee et al. [32] and Ibrahim et al. [41] using *Penicillium* sp. and Actinomycetales bacterium strains. Interestingly, not all silver-resistant isolates were capable of AgNP synthesis, whereas some non-resistant isolates demonstrated this ability, indicating that nanoparticle biosynthesis is not directly correlated with silver resistance, consistent with previous reports [54, 55].

A strong surface plasmon resonance (SPR) peak appearing in the range of 370–460 nm is characteristic of AgNP formation in the UV–Vis spectrum [56]. Variations in color intensity and UV–Vis spectra suggest that the biosynthetic capacity is strain-dependent, likely reflecting differences in metabolic activity. The use of cell-free extracts (CFE) was favored over biomass-based approaches due to the challenges associated with complex media components and nanoparticle recovery from cells [5, 57]. The selected synthesis conditions, including 1 mM AgNO₃, provided efficient and reproducible biosynthesis of AgNPs [41, 56].

The three most active isolates were actinomycetes that produced the highest AgNPs concentrations. βC1, and βB1 were identified as *Streptomyces* sp., while βA15 was *Streptomyces humidus*. The variation in λ_max_ values among the different isolates is likely attributable to differences in the size, shape, degree of aggregation, and surface capping of the biosynthesized AgNPs, which are influenced by isolate-specific biomolecules involved in nanoparticle synthesis and stabilization. Among the tested preparation methods, freeze/thaw produced slightly higher absorbance peaks, whereas soaking provided comparable UV–Vis profiles. However, method selection was not based solely on absorbance intensity. Soaking was selected for subsequent experiments because it provided a simple, practical, and reproducible approach with lower cost and fewer processing steps [57–62]. Sonication appeared less suitable under the conditions used in this study, possibly due to effects on biomolecule functionality.

Regarding the purification of the biosynthesized AgNPs, the most efficient method was selected based on the persistence of the characteristic absorbance peak and the highest concentration. Although dialysis resulted in higher nanoparticle concentrations, shifts in λ_max_ and reduced color intensity indicated possible alterations in nanoparticle properties. Shahzad et al. [56] synthesized Ag NPs with average size of 68 nm and the solution turned to pale yellow after purification. Consequently, cooling centrifugation for 90 min was selected as an optimal purification method, consistent with Iravani [10] and Srikar et al. [63].

The antimicrobial activity of the biosynthesized AgNPs was tested against three microorganisms representing Gram-negative bacteria (*Escherichia coli*), Gram-positive bacteria (*Staphylococcus aureus*), and a fungal pathogen (*Candida albicans*). The antimicrobial activity of the biosynthesized AgNPs was greater than that of commercial AgNPs, as the MIC and MBC values were lower than those of the commercial AgNPs [64]. Greater antimicrobial activity of biosynthesized AgNPs was reported by Gahlawat and Choudhury [6]. Differences in susceptibility between Gram-positive and Gram-negative bacteria were observed, although such trends vary across studies. Gram-positive *S. aureus* was more sensitive to AgNPs although it has a thicker peptidoglycan layer compared to Gram-negative *E. coli.* Al-Dhabi et al. [65] also revealed that *S. aureus* was more sensitive to AgNPs than *E. coli*. In contrast Sayed et al. [64] found that *E. coli* was more sensitive to AgNPs than *S. aureus.* Antimicrobial mechanisms include membrane disruption, the accumulation of AgNPs, and the destruction of intracellular proteins [66], as well as the formation of ROS, which impairs respiration and causes cell death [67]. The observed antimicrobial activity is associated with the biosynthesized nanoparticle preparation; however, because an Ag⁺-only control was not included, the relative contributions of nanoparticles and residual silver ions cannot be distinguished.

In this study, antimicrobial activity was not strongly influenced by the CFE preparation method. The AgNPs, synthesized by soaking CFE of *Bacillus mojavensis* BTCB15, demonstrated inhibition zones ranging from 1 to 6 mm against *E. coli* BTCB03, *Klebsiella pneumoniae* BTCB04, and *Acinetobacter* sp [68]. Additionally, *Acinetobacter calcoaceticus* soaking CFE exhibited antibacterial activity against *P. aeruginosa* and *A. baumannii*, with inhibition zones ranging from 0.2 to 0.6 mm [69].

Although qualitative analysis did not detect carbohydrates, reducing sugars, or phenols in the CFE of the three strains, these substances may be present at concentrations below the detection limit. Quantitative analysis revealed varying concentrations of phenols, nitrate reductase, proteins, DNA, and RNA. The CFE of βC1 exhibited relatively higher concentrations of proteins, DNA, and RNA, along with measurable nitrate reductase activity, which was associated with the formation of AgNPs displaying enhanced colloidal stability (zeta potential: −37.3 ± 6.76 mV). Nithyaja et al. [70] and Kasyanenko et al. [71] used DNA to synthesize stable AgNPs and found that the nanoparticles were attached to N-7 guanine and phosphate base pairs. Polysaccharides, proteins, and enzymes in biological sources are widely reported to contribute to the reduction of metal ions and stabilization of nanoparticles [72].

Peaks corresponding to O–H, N–H, C-H, C = C, and C ≡ C, stretching vibrations observed in FTIR analysis of the dried CFE samples of the three strains indicate the presence and bending of alcohols, phenols, amino functional groups of proteins, aromatic compounds, alkenes, and alkyne. Manivasagan et al. [73] and Eid et al. [74] identified the presence of alkynes, alkene, and amide on the surface of AgNPs synthesized by *Nocardiopsis* sp. and *Streptomyces laurentii*, respectively. Stretching vibrations associated with halo components (Cl-I) and sulfur-containing proteins (C ≡ N) were detected in the CFE of *Streptomyces humidus* (βA15). Sulfur-containing biomolecules such as thiol and thiocyanate may act as a reducing agent in AgNPs biosynthesis as reported by Hussain et al. [75].

Biochemical assays confirmed the presence of nitrate reductase activity in the CFE, and molecular analysis detected the *napA* gene (385 bp amplicon) in all three strains, indicating the genetic potential for periplasmic nitrate reductase production. Similar observations have been reported in Khalaf [76], who identified *napA* in *Pseudomonas aeruginosa* in the context of AgNP biosynthesis. While these findings suggest that nitrate reductase may contribute to the reduction of Ag^⁺^ ions during nanoparticle synthesis, the current evidence remains indirect and does not establish a definitive mechanistic role. Other biomolecules present in the CFE are also likely to participate in the reduction and stabilization processes. Comparable interpretations have been reported by Karthik et al. [77] in *Streptomyces* sp. LK3. Although nitrate reductase activity was not detected in the CFE of the βA15 isolate, the AgNPs synthesized using this extract showed antimicrobial activity comparable to that of the two other AgNPs synthesized using CFEs with detectable nitrate reductase activity. The CFE of βA15 also exhibited the highest concentration of phenols, which may contribute to the reduction of Ag⁺ ions and the stabilization of AgNPs. This supports the possibility of multiple, parallel mechanisms of nanoparticle biosynthesis.

Nanometrological techniques such as UV-Vis spectrophotometry, XRD, FTIR, Zeta sizer, HR-TEM, and AFM were used to fully characterize the biosynthesized AgNPs. The XRD pattern of the biosynthesized AgNPs showed Bragg’s diffraction peaks corresponding to the (111), (200), (220), (311), and (222) planes of the face-centered cubic crystal structure of silver, confirming the crystalline nature of the synthesized nanoparticles. These results are consistent with those reported by Sanjenbam et al. [78] using Streptomyces sp. VITPK1.” The FTIR spectrum confirmed the presence of various functional groups on the synthesized AgNPs including alcoholic, carboxylic, and aromatic groups that may have a role in the capping and stabilization of AgNPs as represented by Sadeghi and Gholamhoseinpoor [79]. FTIR analysis revealed the existence of alkene and amine, and these results are similar to those of Manivasagan et al. [73] and Eid et al. [74] who revealed the existence of alkynes, alkene, and amide on the surface of AgNPs synthesized by *Streptomyces laurentii* and *Nocardiopsis* sp, respectively. TEM and AFM confirmed the formation of predominantly small, spherical to irregular AgNPs with sizes ranging from 5–52 nm. The observed morphology and size distribution are consistent with effective microbial biosynthesis and agree with previous reports for *Streptomyces*-mediated AgNP synthesis [80]. The zeta potential value ranged between 22 and 37 mV as the higher negative or positive value of zeta potential indicates more stable colloidal suspension [81, 82]. This is attributed to electrostatic repulsive forces that reduce nanoparticle aggregation and contribute to colloidal stability. Such stability may facilitate interactions between AgNPs and microbial cells, thereby supporting their biological activity [83].

## Conclusions

In conclusion, bacteria and actinomycetes isolated from a gold mine in Marsa Allam, Egypt exhibited diverse capabilities for AgNP biosynthesis, consistent with their origin from a metal-rich environment. The use of cell-free extracts provides a simple and efficient approach for AgNP synthesis, although purification methods can influence nanoparticle characteristics. Among the most active isolates are two *Streptomyces* sp. and one *Streptomyces humidus* The biosynthesized AgNPs demonstrate potent antimicrobial activity against clinical pathogens, including *E. coli* (NCMB 11943), *S. aureus* (ATCC 25923), and *C. albicans* (ATCC 10231), in comparison to commercial AgNPs. While nitrate reductase may contribute to Ag⁺ reduction, the process is likely mediated by multiple biomolecules, including proteins and phenolic compounds. Overall, gold mine–derived actinomycetes represent promising candidates for biosynthesis of stable and well-characterized AgNPs.

## Data Availability

The datasets generated and/or analyzed during the current study are available from the corresponding author upon reasonable request ( [heba_taher@science.suez.edu.eg](mailto: heba_taher@science.suez.edu.eg) ).
